# A Method for the Acute and Rapid Degradation of Endogenous Proteins

**DOI:** 10.1016/j.cell.2017.10.033

**Published:** 2017-12-14

**Authors:** Dean Clift, William A. McEwan, Larisa I. Labzin, Vera Konieczny, Binyam Mogessie, Leo C. James, Melina Schuh

**Affiliations:** 1 Medical Research Council, Laboratory of Molecular Biology, Cambridge CB2 0QH, UK; 2 Max Planck Institute for Biophysical Chemistry, 37077 Göttingen, Germany

**Keywords:** protein degradation, protein knockdown, cell division, meiosis, primary cells, oocytes, macrophages, antibodies, RNAi, CRISPR/Cas9

## Abstract

Methods for the targeted disruption of protein function have revolutionized science and greatly expedited the systematic characterization of genes. Two main approaches are currently used to disrupt protein function: DNA knockout and RNA interference, which act at the genome and mRNA level, respectively. A method that directly alters endogenous protein levels is currently not available. Here, we present Trim-Away, a technique to degrade endogenous proteins acutely in mammalian cells without prior modification of the genome or mRNA. Trim-Away harnesses the cellular protein degradation machinery to remove unmodified native proteins within minutes of application. This rapidity minimizes the risk that phenotypes are compensated and that secondary, non-specific defects accumulate over time. Because Trim-Away utilizes antibodies, it can be applied to a wide range of target proteins using off-the-shelf reagents. Trim-Away allows the study of protein function in diverse cell types, including non-dividing primary cells where genome- and RNA-targeting methods are limited.

## Highlights

• Trim-Away is a widely applicable method to degrade endogenous proteins
• Target proteins do not need to be modified before degradation
• Proteins are degraded within minutes of application
• Trim-Away allows efficient protein depletion in primary human cells

## Graphical Abstract

**Figure:**
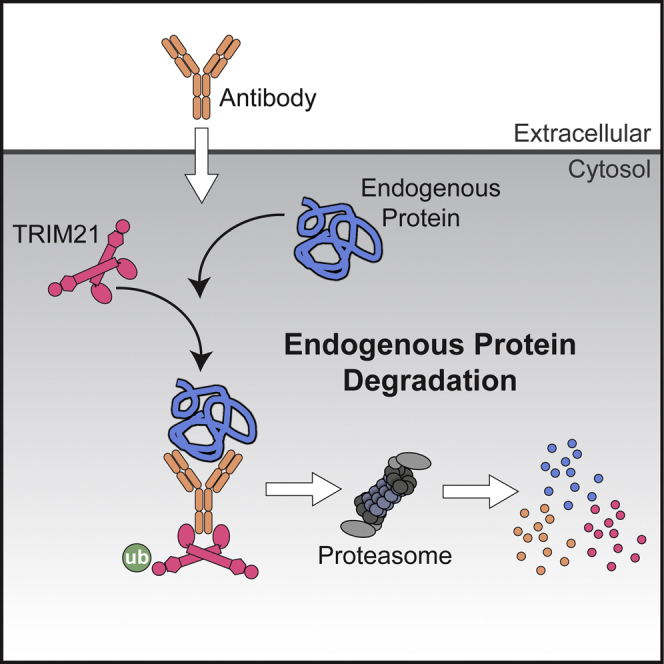

## Introduction

Interfering with protein expression is a powerful strategy to investigate the function of a protein. Traditionally, DNA-modifying methods have been used to knockout proteins on the gene level (Capecchi, 1989), an approach that has had a recent resurgence with the emergence of CRISPR/Cas9 technology (Doudna and Charpentier, 2014). RNA-targeting methods such as RNAi are also widely used to knockdown expression of a protein by destroying the mRNA (Elbashir et al., 2001). However, in both approaches, protein depletion is indirect and dependent on the inherent turnover of the protein. Consequently, long-lived proteins take more time to deplete, or may be resistant to DNA- and RNA-targeting depletion methods altogether (Jansen et al., 2007, Smoak et al., 2016). The long time frame that is required for protein depletion with these methods also means that cells may have enough time to activate compensatory mechanisms, which may mask phenotypes (Damke et al., 1995, Rossi et al., 2015). Furthermore, it is often impossible to determine whether or not a phenotype is an indirect consequence of an earlier defect. In an attempt to overcome these problems, several methods have been developed for conditional protein inactivation. However, these methods require that either the protein of interest is first modified (Banaszynski et al., 2006, Caussinus et al., 2011, Neklesa et al., 2011, Nishimura et al., 2009, Robinson et al., 2010, Tachibana-Konwalski et al., 2010) or can only be applied to a very small number of proteins (Deshaies, 2015, Fan et al., 2014). Chemical inhibitors can be equally problematic as they are limited to druggable proteins and prone to off-target effects (Mayer, 2003). A widely applicable protein depletion method that acts exclusively at the protein level is currently lacking. Such a method would not only allow the acute depletion of endogenous proteins, but also the study of protein function in non-dividing primary cells in which DNA-targeting must be done at the whole animal level and RNA-targeting cannot deplete stable proteins.

We sought to develop a truly posttranslational protein depletion method based on protein targeting by antibodies. Antibodies bind to proteins with high affinity and specificity; they are widely available commercially and can be produced for almost any protein with relative ease. Antibodies are therefore ideal as the basis of a protein-targeting method. Antibodies have been used previously to interfere with protein function (Morgan and Roth, 1988). However, this requires that the antibody binds to an epitope that blocks the function of a protein and can effectively and stoichiometrically compete with endogenous ligands. This method of inhibition is therefore only applicable to a very limited number of proteins. Instead, we aimed to develop a universally applicable method that would allow us to target any antibody-bound protein for degradation.

Antibody-bound pathogens can be recognized by the cytosolic antibody receptor, TRIM21 (Mallery et al., 2010). TRIM21 is an E3 ubiquitin ligase that binds with high affinity to the Fc domain of antibodies (James et al., 2007). TRIM21 is widely expressed in diverse cell types and tissues, which is a necessary requirement of its physiological role (Yoshimi et al., 2009). During infection, TRIM21 recruits the ubiquitin-proteasome system to antibody-bound pathogens, leading to their destruction (Mallery et al., 2010). TRIM21 causes degradation of diverse pathogens including RNA and DNA viruses (Watkinson et al., 2015), bacteria (McEwan et al., 2013), and the proteopathic agent Tau (McEwan et al., 2017). Both the proteasome and the AAA ATPase VCP/p97 have been identified as important co-factors for degradation, but their requirement differs between substrates (Hauler et al., 2012).

The E2 enzymes Ube2W and Ube2N/2V2 have also been implicated in TRIM21-mediated degradation (Fletcher et al., 2015). These enzymes act sequentially on TRIM21 itself, first mono-ubiquitinating it (Ube2W) and then extending from this a K63-ubiquitin chain (Ube2N/2V2). TRIM21 is also modified with K48-chains but the E2 enzyme(s) involved are unknown, as is the functional importance of this modification. TRIM21 has not been reported to modify the pathogen or the pathogen bound antibodies, but it is unclear if such modifications have escaped detection or if auto-ubiquitination of TRIM21 is sufficient for pathogen destruction. TRIM21 has also been reported to interact with several other proteins including Skp2, DAXX, IRF-3, IRF-5, IRF-8, DDX41, and SQSTM1 (James, 2014). However, most of these interactions have been detected based on immunoprecipitation, which can deliver false positives because of direct binding of TRIM21 to the antibodies used in the assay.

In this study, we repurposed TRIM21 to establish a method to degrade endogenous proteins that we called Trim-Away. Trim-Away allows the study of protein function in various cell types, including non-dividing primary cells where genome- and RNA-targeting methods are not well suited. Trim-Away degrades proteins within minutes of application, making it suitable to investigate the function of a protein long after it has formed and at all stages of a cell’s life, cycle, or differentiation. Acute protein disruption assays, which in the past have required complex genetics and a lot of time, can now be done by Trim-Away within hours. Trim-Away therefore forms the basis for the systematic stage-specific analysis of protein function.

## Results

### Principle of Protein Degradation by Trim-Away

**Figure 1:**
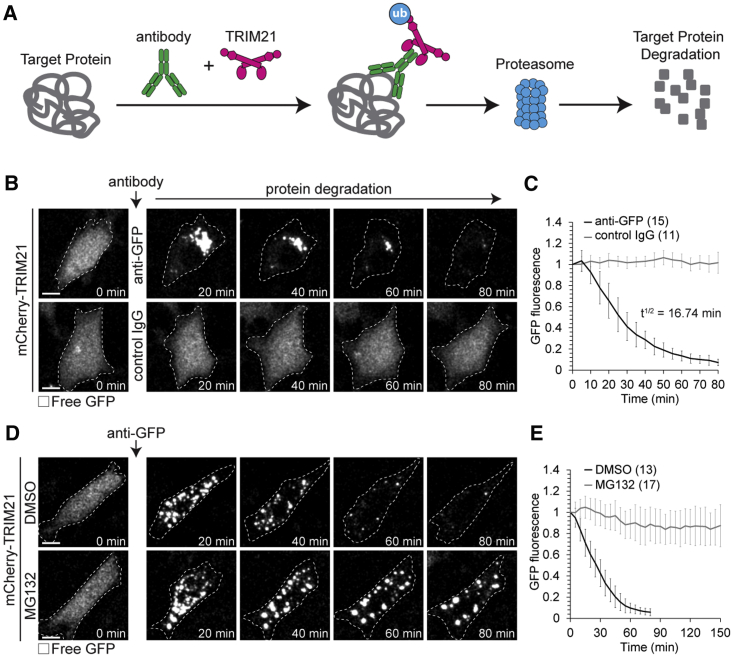
Acute Degradation of Proteins by Trim-Away (A) Schematic of Trim-Away approach. (B–E) NIH 3T3 cells overexpressing mCherry-TRIM21 (not shown) and free GFP (greys) were microinjected with anti-GFP antibody or control IgG (B and C) or treated with DMSO or MG132 and microinjected with anti-GFP antibody (D and E). Time shows minutes (min) from antibody microinjection; 0 min is just before antibody microinjection. Dashed line outlines cell. Scale bars, 10 μm. Error bars show SD. Number of cells is specified in brackets. Data from three (C and E) independent experiments. See also Figures S1 and Figure S3, Figure S4, Figure S5, Figure S6, Figure S7 and Movie S1.

We reasoned that the antibody receptor and ubiquitin ligase TRIM21 could be used as a tool to drive the degradation of endogenous proteins by using a 3-step strategy: first, the introduction of exogenous TRIM21; second, the introduction of an antibody against the protein of interest; and third, TRIM21-mediated ubiquitination followed by degradation of the antibody-bound protein of interest (Figure 1A). As we outline in detail below, this strategy, which we called “Trim-Away,” is ideally suited for the acute and rapid degradation of endogenous proteins in both individual cells as well as bulk cell populations.

**Figure S1:**
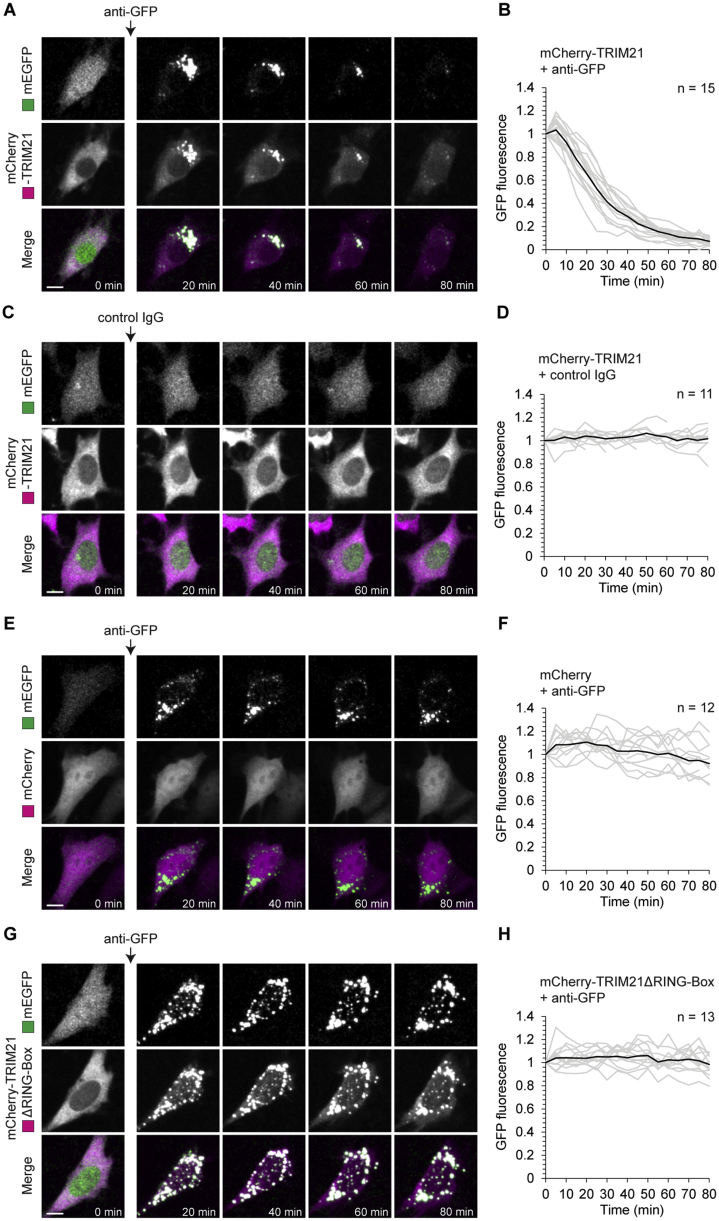
Trim-Away of GFP in NIH 3T3 Cells, Related to Figure 1 NIH 3T3 cells expressing free GFP (green) and either mCherry-TRIM21 (A-D), mCherry (E and F) or mCherry-TRIM21ΔRING-Box (G and H) (magenta) were microinjected with either anti-GFP antibody (A, B and E-H) or control IgG (C and D). Time shows minutes (min) from antibody microinjection; 0 min is just before antibody microinjection. Scale bars, 10 μm. Graphs show plots for individual cells (gray) and mean value (black). Data from three independent experiments (B, D, F and H). See also Movie S1.

To test the Trim-Away strategy, we first set up a proof of principle experiment in mammalian cell culture. NIH 3T3 cells overexpressing mCherry-TRIM21 and free GFP were microinjected with anti-GFP antibody (Figure 1B). Remarkably, GFP was rapidly degraded following microinjection with a half-life of just 16 min (Figures 1B, 1C, and S3A; Movie S1). mCherry-TRIM21 colocalized with GFP during degradation, consistent with TRIM21 recruitment to GFP via the anti-GFP antibody (Figures S1A and S1B; Movie S1). GFP aggregated quickly at the site of antibody microinjection, likely because of the high local concentration of anti-GFP antibody (Figures 1B and S1A). Degradation was specifically due to targeting of GFP by anti-GFP antibody, because microinjection of a non-specific control IgG did not cause GFP degradation in mCherry-TRIM21-overexpressing cells (Figures 1B, 1C, and S1A–S1D). The degradation of GFP was dependent on TRIM21 overexpression, because anti-GFP antibody failed to trigger GFP degradation in cells that overexpressed mCherry instead of mCherry-TRIM21 (Figures S1E and S1F). The E3 ubiquitin ligase activity of TRIM21 was required for degradation, because a truncated form of TRIM21 lacking the RING E3 ubiquitin ligase and B-Box domains (mCherry-TRIM21ΔRING-Box) efficiently colocalized with GFP following anti-GFP antibody microinjection, but failed to cause GFP degradation (Figures S1G and S1H).

To test if protein degradation by Trim-Away is mediated by the proteasome consistent with the known mechanism of TRIM21 function, we treated cells with the proteasome inhibitor MG132. Strikingly, MG132 treatment prevented the degradation of GFP following anti-GFP antibody microinjection into mCherry-TRIM21-overexpressing cells (Figures 1D and 1E). Therefore, protein degradation by Trim-Away relies on the ubiquitin-proteasome pathway (Figure 1A).

### Trim-Away Can Be Used to Degrade Proteins in Primary Cells

We next asked if Trim-Away could be applied to post-mitotic primary cells. We chose mammalian oocytes because they are transcriptionally silent, which precludes protein disruption by direct genome editing. Moreover, RNAi is inefficient in these cells due to large amounts of stored proteins (Clift and Schuh, 2013, Pfender et al., 2015). We first tested if TRIM21 overexpression has any influence on oocyte meiosis. Oocytes that overexpressed TRIM21 progressed through meiosis with similar efficiency and timing as control oocytes: neither the rate nor the timing of nuclear envelope breakdown or anaphase was significantly different (Figures S6A–S6C, S6E, and S6F). Also spindle morphology during meiosis I and meiosis II was not perturbed (Figures S6A, S6D, and S6G). Thus, TRIM21 overexpression does not perturb oocyte meiosis.

**Figure 2:**
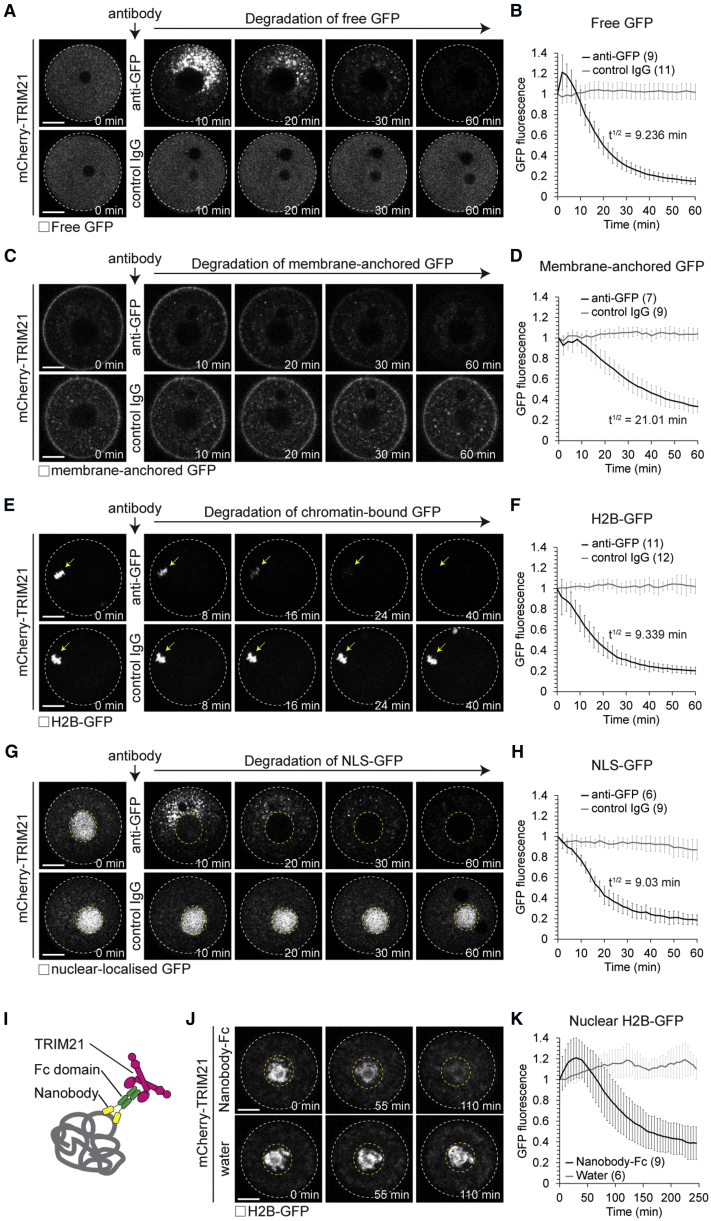
Trim-Away Degrades Diverse Cellular Substrates (A–H) Oocytes overexpressing mCherry-TRIM21 (not shown) and either free GFP (A and B), membrane-anchored GFP (C and D), H2B-GFP (E and F), or NLS-GFP (G and H) were microinjected with either anti-GFP antibody or control IgG. Time shows minutes (min) from antibody microinjection, 0 min is just before antibody microinjection. (I) Schematic of nanobody-Fc fusion approach. (J and K) Prophase-arrested oocytes expressing H2B-GFP were microinjected with either mRNA for mCherry-TRIM21 or mRNA for mCherry-TRIM21 and anti-GFP nanobody-Fc fusion protein. (J) Representative examples and (K) quantification. Time shows minutes (min) from start of imaging. White dashed line outlines oocyte. Yellow arrow shows H2B-GFP. Yellow dashed line outlines nucleus. Scale bars, 20 μm. Error bars show SD. Number of oocytes is specified in brackets. Data from three (B) or two (D, F, H, and K) independent experiments. See also Figure S2, Figure S3, Figure S4, Figure S5, Figure S6, Figure S7.

**Figure S2:**
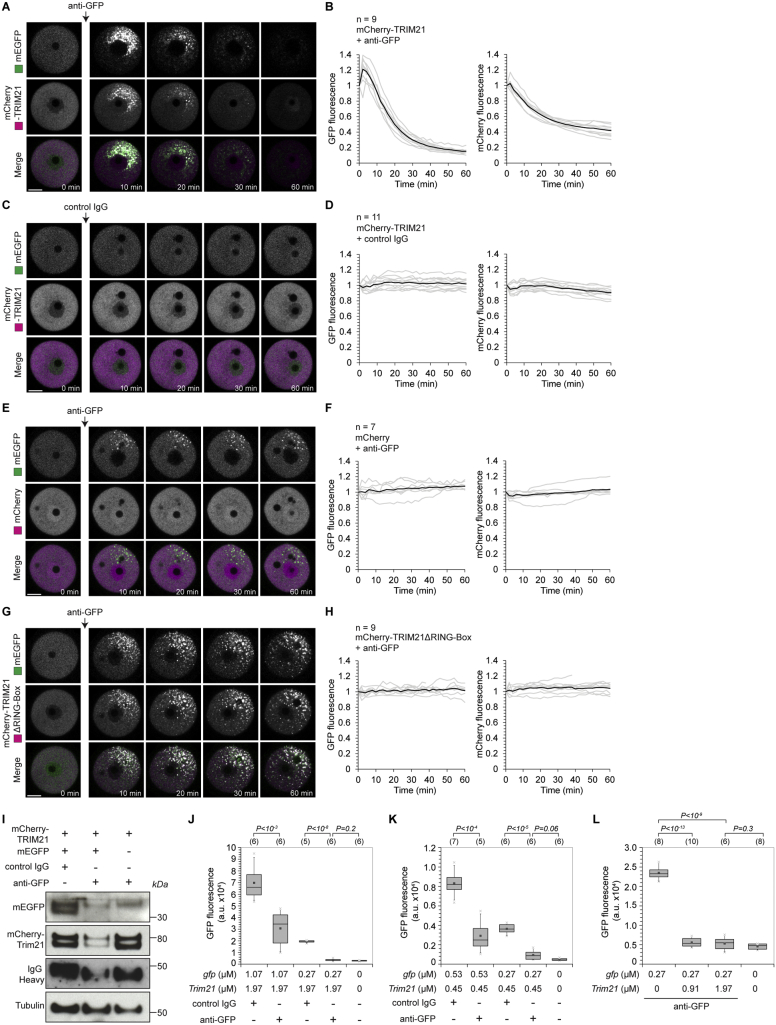
Trim-Away of GFP in Mouse Oocytes, Related to Figure 2 (A–H) Oocytes expressing free GFP (green) and either mCherry-TRIM21 (A-D), mCherry (E and F) or mCherry-TRIM21ΔRING-Box (G and H) (magenta) were microinjected with either anti-GFP antibody (A, B and E-H) or control IgG (C and D). Time shows minutes (min) from antibody microinjection, 0 min is just before antibody microinjection. Scale bars, 20 μm. Graphs show plots for individual oocytes (gray) and mean value (black). Data from two independent experiments (B, D, F and H). (I) Oocytes expressing either mCherry-TRIM21 and free GFP, or mCherry-TRIM21 alone were microinjected with control IgG or anti-GFP antibodies and whole cell lysates harvested 1 hour later for immunoblotting. (J and K) Oocytes were microinjected with varying amounts of *gfp* or *mCherry-Trim21* mRNA, incubated for 3-5 hours to allow GFP and mCherry-TRIM21 protein expression, microinjected with either PBS, control IgG or ant-GFP antibodies and GFP fluorescence determined 2 hours later by microscopy. Number of oocytes specified in brackets. *P value*s were calculated with Student’s t test.

We then performed a similar proof-of-principle GFP degradation experiment as described above in isolated mouse oocytes. Microinjected anti-GFP antibody triggered rapid GFP degradation in oocytes overexpressing mCherry-TRIM21. In contrast, control IgG had no effect on GFP protein levels (Figures 2A and 2B). As in NIH 3T3 cells, mCherry-TRIM21 colocalized with GFP during GFP degradation (Figure S2A), and GFP degradation was dependent on TRIM21’s ubiquitin ligase activity (Figures S2C–S2H). Before protein degradation, we observed a transient increase in GFP intensity at the site of GFP-antibody microinjection (Figure 2A). This is likely due to a local enrichment of GFP-antibody at the microinjection site, which results in sequestration of GFP from the entire oocyte volume to this region.

We noticed that mCherry-TRIM21 was depleted concomitantly with GFP upon anti-GFP antibody microinjection (Figures S2A and S2B). Western blotting of whole cell extracts confirmed that TRIM21, antibody and target protein are all degraded during Trim-Away (Figure S2I). This is consistent with the proposed mechanism of TRIM21 function and suggests that TRIM21 and antibody levels could become limiting during Trim-Away if the target is in significant molar excess. To investigate this further, we modified relative levels of TRIM21 and GFP and found that TRIM21 needs to be present in excess of the target protein to facilitate complete protein degradation (Figures S2J–S2L).

### Trim-Away Can Target Diverse Cellular Substrates

**Figure S3:**
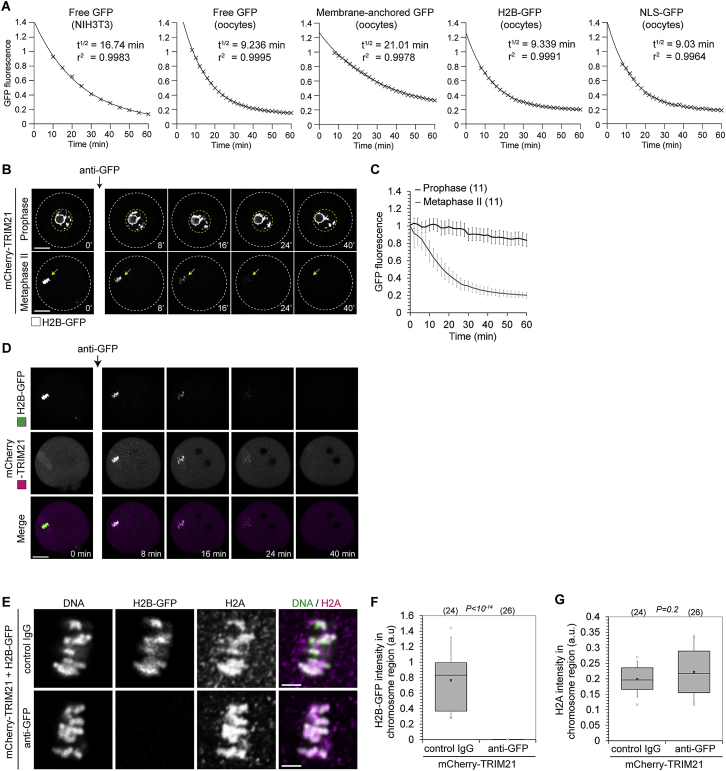
Trim-Away of Diverse Cellular Substrates, Related to Figures 1 and 2 (A) Prism (GraphPad) software was used to fit single phase decay curves to the mean values (black crosses) of GFP fluorescence taken from data shown in Figures 1C, 2B, 2D, 2F, and 2H. T^1/2^ indicates half-life in minutes (min). R^2^ indicates goodness of fit of single phase decay curve. (B and C) Oocytes overexpressing mCherry-TRIM21 (not shown) and H2B-GFP (greys) were arrested in prophase or metaphase of meiosis II and microinjected with anti-GFP antibody. Time shows minutes (min) from antibody microinjection. White dashed lines outline oocytes. Yellow arrows show H2B-GFP. Yellow dashed lines outline nucleus. Scale bars, 20 μm. Error bars show s.d. Number of oocytes in brackets. Data from two independent experiments. (D) Oocytes overexpressing mCherry-TRIM21 (magenta) and H2B-GFP (green) were microinjected with anti-GFP antibody. mCherry-TRIM21 recruitment and H2B-GFP degradation occurs in the chromosome region, suggesting that H2B-GFP is degraded while assembled in nucleosomes. Time minutes (min) from microinjection. Scale bar, 20 μm. (E) Oocytes overexpressing mCherry-TRIM21 and H2B-GFP were microinjected with either control IgG or anti-GFP antibody and fixed 1 hour later for immunofluorescence. DNA stained with Hoechst. Scale bar, 5 μm. (D–G) Trim-Away of H2B-GFP does not cause degradation of H2A. Immunofluorescence images show normal chromosome morphology and H2A localization following H2B-GFP degradation (E). Boxplots of H2B-GFP (F) and H2A (G) intensity in the chromosome region. Degradation of H2B-GFP did not affect the levels of histone H2A on chromosomes, implying that the remaining components of the histone complex were not co-degraded with H2B-GFP. Number of cells in brackets. Data from two independent experiments. *P value*s were calculated with Student’s t test.

A widely applicable protein depletion method should be able to target diverse substrates within the cell. To address this, we localized GFP to different regions of the cell and tested the efficiency of degradation by Trim-Away. GFP containing an N-terminal N-myristoyl and S-palmitoyl motif (membrane-anchored GFP) localized to the plasma membrane and vesicle-like structures in the cytoplasm (Figure 2C); GFP fused to the histone H2B (H2B-GFP) was efficiently incorporated into chromatin (Figure 2E); GFP containing a nuclear localization signal (NLS-GFP) accumulated in the nucleus (Figure 2G). All three different substrates were rapidly degraded by anti-GFP antibody and TRIM21 with similar kinetics to free cytosolic GFP (Figures 2C–2H). Remarkably, the half-life for degradation by Trim-Away was as little as 9 min (Figures 2B, 2D, 2F, 2H and S3A). This is identical to the rate of protein degradation achieved by the auxin-inducible degron system (Holland et al., 2012), but with the advantage that Trim-Away requires no modification of the target protein. Altogether, these data show that Trim-Away can degrade proteins that are localized to different regions of the cell and incorporated into larger protein complexes and cellular structures.

We noticed that NLS-GFP was degraded in the cytoplasm, presumably because NLS-GFP shuttles in and out of the nucleus and can therefore be bound by antibodies in the cytoplasm (Figure 2G). We therefore tested if Trim-Away can also degrade retained nuclear proteins, such as GFP-H2B that is stably associated with chromatin within the nucleus. In contrast to NLS-GFP, GFP-H2B was not degraded when the chromatin was contained within an intact nucleus (Figures S3B and S3C). We reasoned that we might be able to overcome this limitation by fusing the Fc-domain of an antibody to a nanobody. The Fc-nanobody fusion is much smaller in size and should be able to enter the nucleus. Strikingly, H2B-GFP was quickly degraded also inside the nucleus when TRIM21 and the Fc-nanobody fusion were co-expressed (Figures 2I–2K). This demonstrates that Fc-nanobody fusions can be used to degrade proteins inside the nucleus. It also illustrates that the growing number of nanobodies is compatible with Trim-Away when these nanobodies are fused with an Fc-domain.

### Rescue Experiments Confirm Trim-Away Specificity

In principle, it should be possible to degrade any endogenous protein in the cell that can be accessed by antibodies using Trim-Away (Figure 1A). To test this, we decided to target an endogenous protein in mouse oocytes called Eg5. Eg5 is a microtubule motor protein required for proper spindle assembly during mitosis and meiosis (Clift and Schuh, 2015, Mayer et al., 1999, Schuh and Ellenberg, 2007). When the function of Eg5 is disrupted, a monopolar microtubule aster forms and the spindle fails to become bipolar. We chose to Trim-Away Eg5 because we could directly compare the phenotype following Eg5 degradation to that of Eg5 inhibition with the small molecule inhibitor monastrol (Mayer et al., 1999).

**Figure 3:**
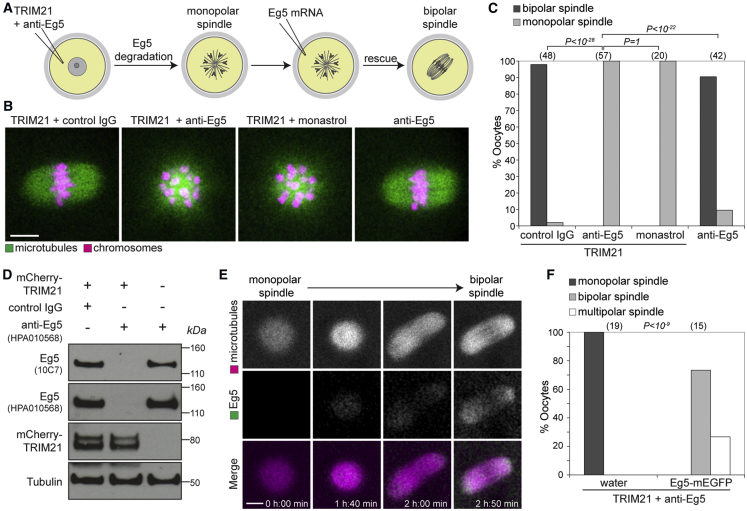
Trim-Away of Endogenous Eg5 Protein (A) Schematic of Eg5 Trim-Away and rescue experiments. (B and C) Oocytes overexpressing TRIM21 and microinjected with either control IgG, anti-Eg5 or treated with monastrol, or oocytes microinjected with anti-Eg5 alone were assessed for spindle morphology. (B) Representative examples and (C) quantification. Microtubules and chromosomes were labeled with mEGFP-Map4 and H2B-mCherry, respectively. (D) Whole oocyte lysates were immunoblotted for the indicated proteins. (E and F) Oocytes overexpressing TRIM21 and microinjected with anti-Eg5 antibody were allowed to form a monopolar spindle and then microinjected with either water or *Eg5-mEGFP* mRNA and scored for spindle morphology. (E) Representative examples and (F) quantification. Microtubules were labeled with mCherry-MAP4. Scale bars, 10 μm. Number of oocytes is specified in brackets. Data from two (D and F) or four (C) independent experiments. P values were calculated with Fisher’s exact test. See also Figures S4, S6, and S7 and Movie S2.

Strikingly, microinjection of anti-Eg5 antibody into oocytes overexpressing TRIM21 caused the formation of monopolar spindles; precisely the phenotype expected if Eg5 is degraded and identical to oocytes treated with the Eg5 inhibitor monastrol (Figures 3A–3C). Neither control IgG microinjection into TRIM21-overexpressing oocytes, nor anti-Eg5 antibody microinjection alone caused monopolar spindles (Figures 3B and 3C), confirming that Eg5 degradation requires both TRIM21 and anti-Eg5 antibody. Eg5 protein was completely degraded as shown by immunoblotting with two different anti-Eg5 antibodies (Figure 3D).

To confirm that the monopolar spindle phenotype was due to Eg5 degradation, we performed a rescue experiment by microinjecting mRNA encoding Eg5-mEGFP into Eg5 Trim-Away oocytes (Figures 3A and 3E). Eg5-mEGFP expression transformed the monopolar spindles back to a bipolar state (Figures 3E and 3F; Movie S2). Eg5-mEGFP can be recognized by anti-Eg5 antibody, but likely rescues because it is expressed in excess. Altogether, these data show that endogenous proteins can be degraded using Trim-Away, and rescue assays can be used to confirm the specificity of a Trim-Away phenotype.

### Trim-Away Is Suitable to Degrade Long-Lived Proteins Acutely

Long-lived intracellular proteins are surprisingly common (Toyama et al., 2013), but studying their function is challenging. The lack of protein turnover means that depletion by RNAi is ineffective. Gene knockouts are also often not suitable, because long-lived proteins are frequently especially important in non-dividing primary cells and essential for viability (Toyama et al., 2013). We reasoned that because Trim-Away acts exclusively at the protein level, it should be suitable to degrade even very long-lived proteins and thereby study their function long after their synthesis.

To test this, we targeted the long-lived Rec8 protein in mouse eggs that were arrested in metaphase of the second meiotic division. Rec8 is part of the cohesin protein complex that mediates sister chromatid cohesion in oocytes from birth until ovulation (Tachibana-Konwalski et al., 2010). Rec8 does not turnover (Tachibana-Konwalski et al., 2010), but remains stably associated with chromosomes for months in mice and possibly decades in humans.

**Figure 4:**
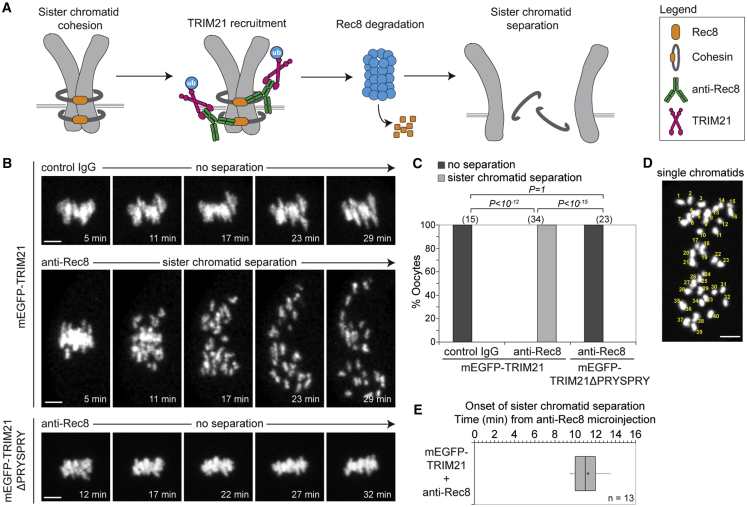
Trim-Away of Long-Lived Rec8 Protein (A) Schematic of Rec8 Trim-Away experiment. (B and C) Eggs overexpressing mEGFP-TRIM21 or mEGFP-TRIM21ΔPRYSPRY (not shown) were microinjected with either control IgG or anti-Rec8 antibody and chromosome morphology (H2B-mCherry; greys) followed by live imaging. (B) Representative examples and (C) quantification. (D and E) Single chromatid count (D) and boxplot of time of onset of sister chromatid separation (E) in eggs overexpressing mEGFP-TRIM21 following microinjection of anti-Rec8 antibody. Scale bars, 5 μm. Time shows minutes (min) from microinjection. Number of eggs in brackets. Data from three (C) or two (E) independent experiments. P values were calculated with Fisher’s exact test. See also Figures S4, S6, and S7 and Movie S3.

We asked if we could use Trim-Away to degrade endogenous Rec8 acutely in metaphase II arrested eggs, long after it has been incorporated into chromosomes. Microinjection of anti-Rec8 antibody (Eijpe et al., 2003) into eggs overexpressing mEGFP-TRIM21 triggered the premature separation of sister chromatids (Figures 4A–4C; Movie S3), producing 40 single chromatids (Figure 4D), which is indicative of Rec8 degradation and complete loss of sister chromatid cohesion (Tachibana-Konwalski et al., 2010). No separation was observed when control IgG was microinjected into mEGFP-TRIM21-overexpressing oocytes or when oocytes overexpressing antibody-binding-deficient TRIM21 (mEGFP-TRIM21ΔPRYSPRY) were microinjected with anti-Rec8 antibody (Figures 4B and 4C). This confirmed that sister chromatid separation was due to Rec8 degradation triggered by TRIM21 recruitment to Rec8 via anti-Rec8 antibody (Figure 4A). Previous functional studies of Rec8 required complex mouse genetics to replace the *Rec8* gene with a version that expresses a TEV protease-cleavable Rec8 protein (Tachibana-Konwalski et al., 2010). The Rec8 Trim-Away experiments described here provide the first evidence that endogenous, unmodified Rec8 protein is responsible for sister chromatid cohesion in mouse eggs. Remarkably, sister chromatids began to separate on average just 11 min after anti-Rec8 antibody microinjection into mEGFP-TRIM21-overexpressing eggs (Figures 4B and 4E), implying Trim-Away can degrade even very long-lived proteins with unprecedented speed.

### Trim-Away Can Be Used to Degrade Specific Protein Variants Selectively

**Figure S4:**
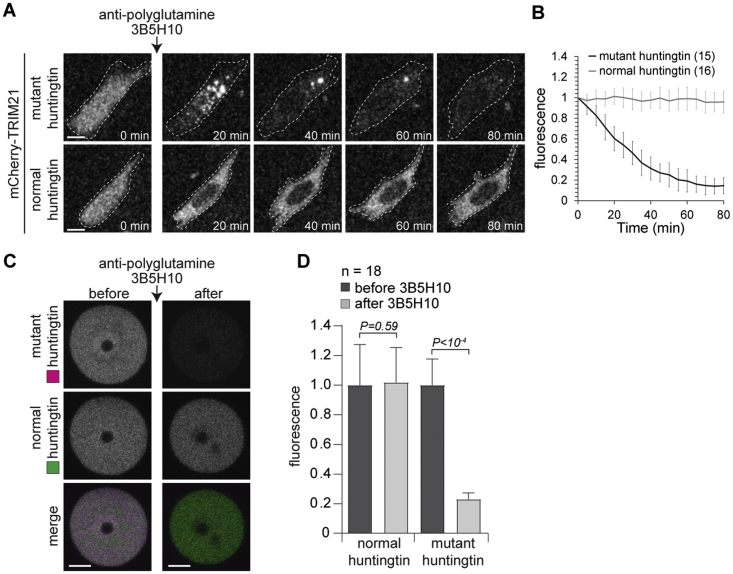
Selective Trim-Away of Mutant Huntingtin Protein, Related to Figure 1, Figure 2, Figure 3, Figure 4, Figure 5, Figure 6, Figure 7 (A and B) NIH 3T3 cells overexpressing mCherry-TRIM21 (not shown) and either EGFP-Htt74Q (mutant Huntingtin; greys) or EGFP-Htt23Q (normal huntingtin; greys) were microinjected with 3B5H10 antibody. Time shows minutes (min) from antibody microinjection; 0 min is just before antibody microinjection. Dashed line outlines cell. Scale bars, 10 μm. (C and D) Oocytes co-expressing TRIM21, EGFP-Htt74Q (mutant Huntingtin; magenta) and EGFP-Htt23Q (normal huntingtin; green) were imaged before and 2.5 hours after microinjection of 3B5H10 antibody. Scale bars, 20 μm. Error bars show s.d. Number of cells in brackets in (B); n refers to number of oocytes in (D). Data from two independent experiments. *P value*s were calculated with Student’s t test.

Given that Trim-Away capitalizes on the high specificity of antibodies, it should also be suitable to selectively degrade splice variants, posttranslationally modified or disease-causing protein variants while preserving the healthy protein, as long as antibodies are available that are variant-specific. To test this, we asked if Trim-Away can be used to selectively degrade the disease-causing variant of the protein huntingtin, which causes Huntington’s disease. We co-expressed the normal or the disease-causing variant of huntingtin protein together with mCherry-TRIM21 in NIH 3T3 cells. We then microinjected the cells with an antibody (3B5H10) which specifically binds to the disease-causing variant of huntingtin (Miller et al., 2011) (Figure S4A). The disease-causing variant of huntingtin protein was rapidly degraded following microinjection of the antibody, whereas normal huntingtin protein was preserved (Figures S4A and S4B). Similar results were obtained in oocytes that expressed both the normal and the disease-causing variant of huntingtin simultaneously: the disease-causing variant was degraded, while the normal variant was preserved (Figures S4C and S4D). This demonstrates that Trim-Away can be used to degrade specific protein variants selectively.

### Antibody Electroporation Allows Trim-Away in Bulk Cell Populations

While microinjection is suitable for studying individual cells, most assays require the manipulation of bulk cell populations. We therefore aimed to develop a method of antibody and TRIM21 delivery that would make Trim-Away applicable to large numbers of cells simultaneously. Recent work has shown that antibodies can be delivered into the cytoplasm by electroporation (Freund et al., 2013), suggesting that antibody electroporation could be used to apply Trim-Away to large cell numbers.

**Figure 5:**
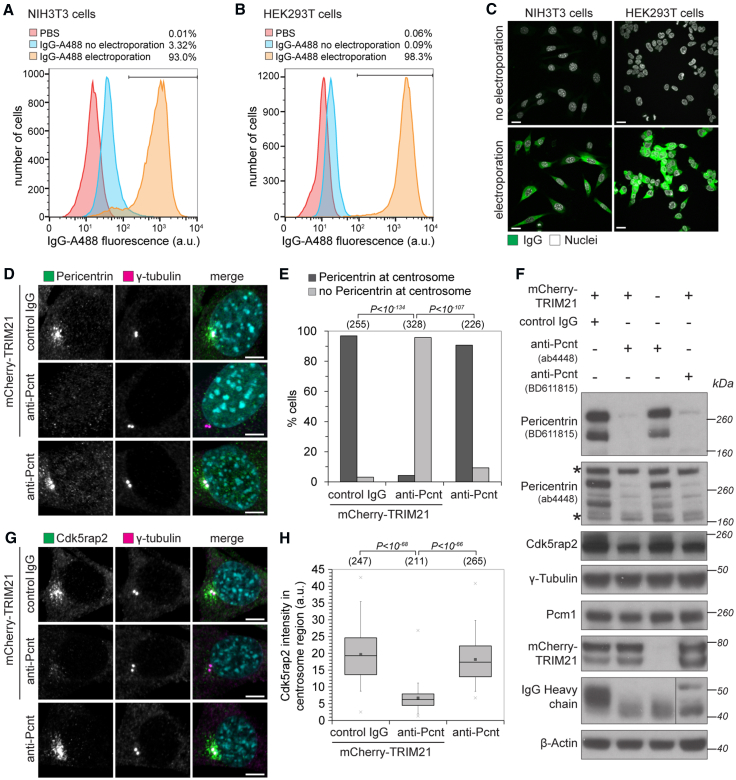
Trim-Away of Pericentrin by Antibody Electroporation (A–C) NIH 3T3 and HEK293T cells were electroporated with Alexa Fluor 488-labeled IgG and analyzed 3 hr later by flow cytometry (A and B) or fixed 16 hr later and analyzed by microscopy (C). At least 2,000 cells were counted for each condition. Percentages correspond to IgG-positive cells falling within the gate drawn. Scale bars, 10 μm. (D–H) NIH 3T3 and NIH 3T3-mCherry-TRIM21 cell lines were electroporated with control IgG or anti-pericentrin antibodies and analyzed 16 hr later for pericentrin (D and E) and Cdk5rap2 localization (G and H). Number of cells in brackets. Scale bars, 5 μm. DNA stained with Hoechst. (F) Cell lysates were immunoblotted for the indicated proteins. Asterisks show non-specific bands not degraded by Trim-Away. Data from two independent experiments (E and H). P values were calculated with Fisher’s exact (E) or Student’s t test (H). See also Figure S4, Figure S5, Figure S6, Figure S7.

**Figure S5:**
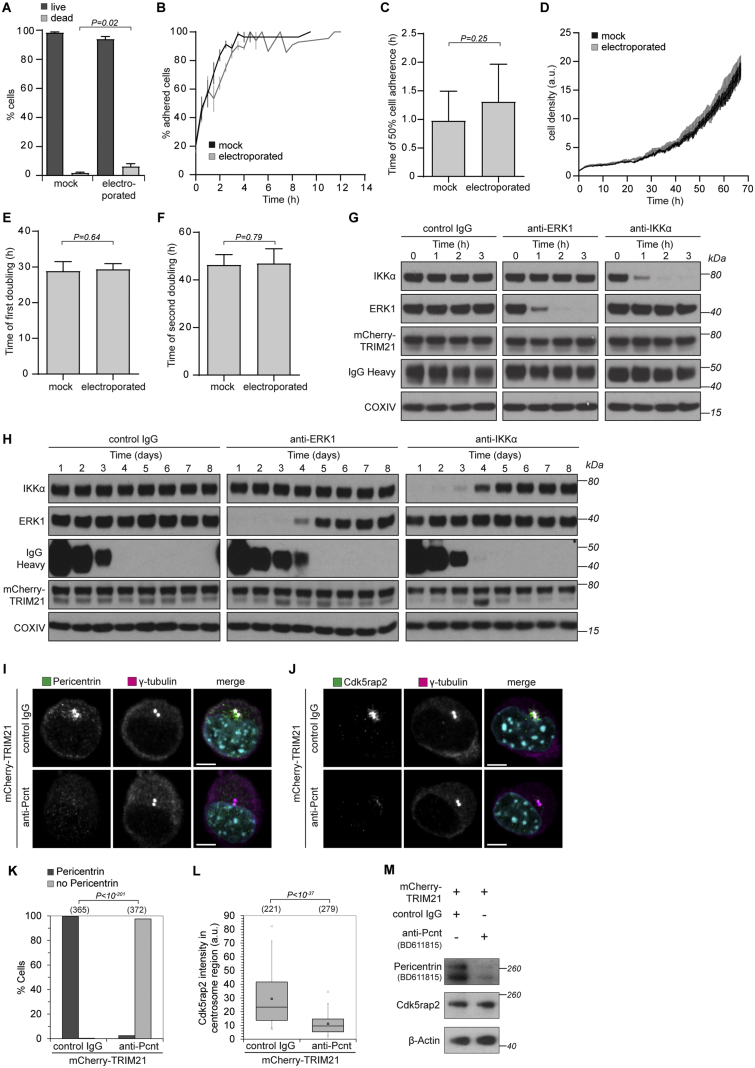
Time Frame for Protein Depletion by Trim-Away, Related to Figure 5 (A–F) NIH 3T3-mCherry-TRIM21 cells were taken up into the Neon Pipette Tip and either electroporated or not (mock). Percentage of dead cells was determined using the trypan blue exclusion assay (A). For long-term analysis of cellular behavior cells were imaged every 15 min for 70 h following electroporation (B-F). For the growth curves cell density was normalized to the initial density once cells had adhered (D). For adherence analysis the percentage of spread cells was quantified in each frame (B and C). Doubling times (E and F) were calculated taking the 5 h time point as reference, because cells were fully adhered then. Data from three independent experiments. Error bars show s.d. *P value*s were calculated with Student’s t test. See also Movie S4. (G and H) HEK293T-mCherry-TRIM21 cells were electroporated with either control IgG, anti-ERK1 or anti-IKKα antibodies and whole cell lysates harvested at the indicated times after electroporation for immunoblotting. (I–M) NIH 3T3-mCherry-TRIM21 cells were electroporated with control IgG or anti-Pericentrin antibody (BD611815) and 3 hours later analyzed for Pericentrin (I and K) or Cdk5rap2 (J and L) localization or cell lysates were immunoblotted for the indicated proteins (M). Merge also shows DNA stained with Hoechst. Number of cells in brackets. Scale bars, 5 μm. Data from two independent experiments. *P value*s were calculated with Fisher’s exact (K) or Student’s t test (L).

Thus, we sought to develop an optimized antibody electroporation protocol. To determine the most efficient electroporation conditions, we used fluorescently labeled antibodies and quantified the number of fluorescent cells upon electroporation (Figures 5A–5C; STAR Methods). The optimized electroporation protocol resulted in highly efficient delivery of antibody into the cytoplasm without cell death (Figures 5A–5C and S5A; STAR Methods). This method of antibody delivery is also compatible with the rapid observation of phenotypes: live imaging revealed that adherent cells started to re-adhere within 30 min of electroporation, with adherence completed by 4 hr (Figures S5B and S5C; Movie S4). Electroporated cells showed no evidence of stress or damage and rapidly re-entered the cell cycle with similar division rates as non-electroporated cells (Figures S5D–S5F; Movie S4).

**Figure S6:**
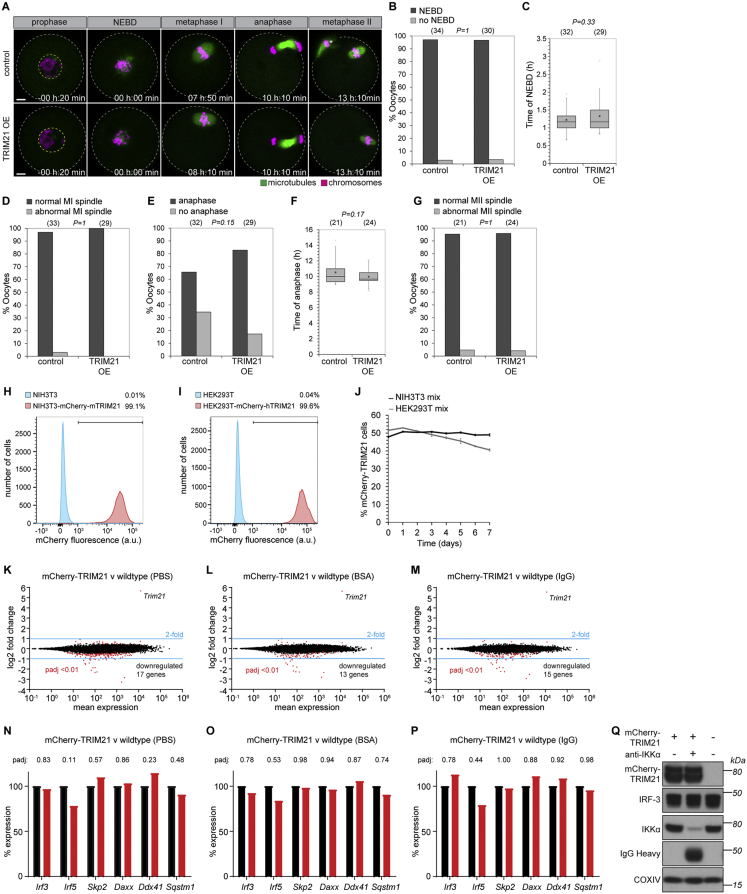
TRIM21 Overexpression Does Not Perturb Cells, Related to Figure 1, Figure 2, Figure 3, Figure 4, Figure 5, Figure 6, Figure 7 (A) Oocytes microinjected with either water (control) or *Trim21* mRNA (TRIM21 OE) were imaged for sixteen hours following release from prophase arrest. Microtubules and chromosomes were labeled with mEGFP-Map4 and H2B-mCherry respectively. Time shows hours and minutes (h:min) from nuclear envelope breakdown (NEBD). Scale bars, 10 μm. White dashed line outlines oocyte. Yellow dashed line outlines nucleus. (B–G) Key events in meiosis were quantified. Number of oocytes is specified in brackets. *P value*s were calculated with Fisher’s exact test (B, D, E and G) or Student’s t test (C and F). (H and I) NIH 3T3, NIH 3T3-mCherry-mTRIM21, HEK293T and HEK293T-mCherry-hTRIM21 cell lines were analyzed by flow cytometry. At least 2000 cells were counted for each condition. Percentages correspond to mCherry-positive cells falling within the gate drawn. (J) NIH 3T3 and NIH 3T3-mCherry-mTRIM21 cells (NIH 3T3 mix) or HEK293T and HEK293T-mCherry-hTRIM21 cells (HEK293T mix) were mixed 50:50 and percentage mCherry cells analyzed by flow cytometry every 24h for 7 days. At least 10,000 cells counted at each time point. Error bars show s.d. Data from 3 independent replicates. (K–M) Transcriptomes of NIH 3T3-mCherry-mTRIM21 cells were compared to wild-type NIH 3T3 cells by RNA-seq analysis (STAR Methods) following electroporation with PBS (K), BSA (L) or IgG (M). A total of only 8 protein-encoding transcripts were consistently downregulated more than 2-fold in the NIH 3T3-mCherry-mTRIM21 cells (*Kdm5d*, *Ddx3y*, *Eif2s3y*, *Uty*, *Asb4*, *Fat4*, *Papss*2 and *Lama2*), although we cannot rule out the possibility that this is an indirect consequence of lentivirus construct integration rather than a direct consequence of TRIM21 overexpression. (N–P) Relative expression levels of genes encoding proteins reported to be ligands of TRIM21. Taken from RNA sequencing data. *Irf8* was not detected. See also Tables S1, S2, and S3. (Q) HEK293T and HEK293T-mCherry-hTRIM21 cells were electroporated with PBS or anti-IKKα antibody and whole cell lysates harvested 3 hours later for immunoblotting. IRF-3 protein levels are unaffected by TRIM21 overexpression or activation.

We also generated stable NIH 3T3 (mouse) and HEK293T (human) cell lines overexpressing mCherry-TRIM21. These cell lines proliferated at a similar rate to wild-type cells and behaved indistinguishably after multiple passages suggesting that TRIM21 overexpression is stable and nontoxic (Figures S6H–S6J; STAR Methods). We also analyzed the transcriptome of TRIM21-overexpressing and wild-type NIH 3T3 cells either with or without electroporation of a non-targeting antibody, BSA or PBS. The transcriptomes were closely related under all conditions, with the vast majority of genes being expressed at similar levels (Figures S6K–S6M; Tables S1, S2, and S3). Notably, transcripts encoding previously described ligands of TRIM21, IRF-3, IRF-5, Skp2, DAXX, DDX41, and SQSTM1 were not significantly increased or decreased upon overexpression of TRIM21 (Figures S6N–S6P). Consistent with this result, neither TRIM21 overexpression, nor Trim-Away of an endogenous protein caused changes in the protein level of IRF-3 (Figure S6Q). Together, these results suggest that cells overexpressing TRIM21 behave similar to wild-types.

We reasoned that by electroporating antibodies into TRIM21-overexpressing cell lines it should be possible to target endogenous proteins in bulk cell populations for degradation. To test this, we targeted two proteins that are non-essential for cell viability: ERK1, a redundant serine/threonine kinase in the MAPK/ERK signaling cascade (Frémin et al., 2015), and IKKα, a catalytic subunit of the IκB kinase (IKK) complex (Hu et al., 1999, Takeda et al., 1999). Strikingly, both ERK1 and IKKα were depleted within 1–2 hr of antibody electroporation into TRIM21-overexpressing cells, with depletion lasting for 3–4 days (Figures S5G and S5H). The reappearance of ERK1 and IKKα coincided with the depletion of antibody from the cells (Figure S5H). This indicates that antibody availability limits the duration of protein depletion and suggests that the depletion period could be modified by varying the amount of electroporated antibody.

We next tested if Trim-Away using antibody electroporation is compatible with quantitative analysis of cellular phenotypes. To this end, we targeted the centrosomal protein pericentrin in NIH 3T3 cells. Strikingly, electroporation of rabbit polyclonal anti-pericentrin antibody (ab4448) into mCherry-TRIM21-overexpressing cells led to a complete loss of pericentrin signal at the centrosome in ∼95% of cells (Figures 5D and 5E). The high efficiency of pericentrin loss likely reflects the fact that ∼95% of cells receive antibody following electroporation (Figures 5A and 5C) and ∼99% of cells overexpress TRIM21 (Figure S6H). Pericentrin degradation by Trim-Away was confirmed by immunoblotting with two different antibodies (Figure 5F). Pericentrin is proposed to have an important role in the localization of Cdk5rap2 to the centrosome (Chen et al., 2014, Lee and Rhee, 2011). However, previous studies relied on prolonged, indirect depletion of pericentrin by RNAi or gene knockout (Chen et al., 2014, Lee and Rhee, 2011). Thus, it has been impossible to distinguish between a role for pericentrin in Cdk5rap2 recruitment during the course of the centrosome cycle or in maintenance of Cdk5rap2 at the centrosome. We used Trim-Away to address this question and investigate how Cdk5rap2 localization is altered upon acute removal of pericentrin. Repeating Trim-Away and staining for Cdk5rap2 revealed that loss of pericentrin from the centrosome leads to a concomitant loss of centrosomal Cdk5rap2 (Figures 5G and 5H).

To further confirm the specificity of pericentrin degradation, we repeated Trim-Away with a second mouse monoclonal anti-pericentrin antibody (BD611815). Pericentrin Trim-Away with BD611815 also caused pericentrin degradation and Cdk5rap2 mislocalization (Figures 5F and S5I–S5M). Notably, pericentrin degradation and Cdk5rap2 mislocalization was observed just 3 hr post-antibody electroporation (Figures S5I–S5M), further demonstrating that pericentrin is required to maintain Cdk5rap2 at the centrosome. Together, this data shows that Trim-Away is suitable to rapidly degrade endogenous proteins and observe cellular phenotypes in bulk cell populations.

### Trim-Away Can Rapidly Activate Signal Transduction Pathways through Selective Degradation

**Figure 6:**
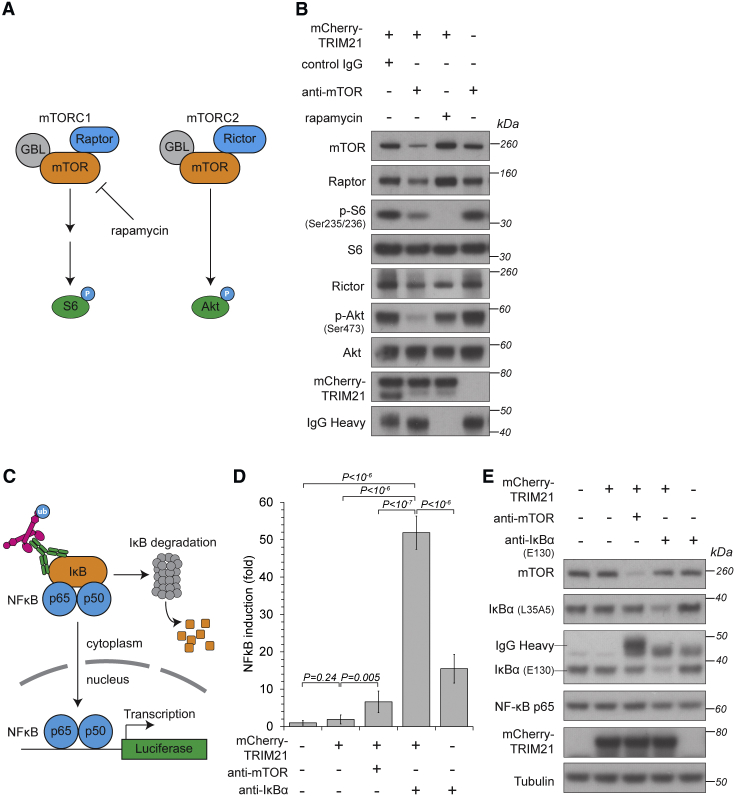
Selective Trim-Away of Signaling Pathway Components (A) Schematic of mTOR function. Rapamycin inhibits only mTORC1. (B) HEK293T and HEK293T-mCherry-TRIM21 cell lines were electroporated with control IgG, anti-mTOR antibody or treated with rapamycin, harvested 5 hr later, and cell lysates immunoblotted for the indicated proteins. (C) Schematic of IκBα Trim-Away experiment. (D and E) HEK293T and HEK293T-mCherry-TRIM21 cell lines transfected with NF-κB-luciferase reporter plasmid were electroporated with PBS, anti-mTOR, or anti-IκBα antibodies and 5 hr later analyzed for luciferase activity (D) or harvested and lysates blotted for the indicated proteins (E). Error bars show SD. Representative examples from two (B) or four (D and E) independent experiments. P values were calculated with Student’s t test. See also Figures S4, S6, and S7.

The above experiments show how Trim-Away can be used to investigate loss-of-function phenotypes. Next, we attempted to use Trim-Away to target proteins involved in active signaling pathways to induce both pathway inactivation and activation. First, we targeted the protein kinase mTOR as it regulates several essential cellular functions (Laplante and Sabatini, 2009) (Figure 6A). Electroporation of anti-mTOR antibody into HEK293T cells overexpressing mCherry-TRIM21 lead to a reduction in mTOR protein levels (Figure 6B). Degradation was not complete, perhaps because a subset of mTOR resides within intracellular compartments inaccessible to antibody (Betz and Hall, 2013). Nonetheless, mTOR degradation by Trim-Away was sufficient to reduce phosphorylation of ribosomal protein 6 at Ser235/236, consistent with a loss of mTORC1 activity and similar to mTORC1 inhibition by rapamycin (Figures 6A and 6B). Importantly, mTOR also functions in a second complex, mTORC2, that induces phosphorylation of Akt. However, this activity cannot be investigated by rapamycin as it is a poor inhibitor of the mTORC2 complex (Sarbassov et al., 2005) (Figure 6A). In contrast, mTORC2 function could be efficiently ablated using Trim-Away. Phosphorylation of Akt at Ser473 was lost upon mTOR degradation by Trim-Away, but only slightly reduced upon rapamycin treatment (Figure 6B).

Next, we attempted to use Trim-Away to induce a change in a transduction pathway that activates rather than inhibits downstream signaling. We chose the nuclear factor κB (NF-κB) pathway as this system is constitutively repressed by the inhibitor IκBα. Upon stimulation, IKK phosphorylates IκBα leading to IκBα ubiquitination and degradation, allowing nuclear translocation of the heterodimeric p65 and p50 complex and transcription of target genes (Gilmore, 2006). Strikingly, electroporation of anti-IκBα antibody into HEK293T cells overexpressing mCherry-TRIM21 triggered both the degradation of IκBα and the substantial induction of NF-κB activity (Figures 6D and 6E). Thus, acute degradation of IκBα by Trim-Away is sufficient to relieve inhibition of the NF-κB pathway and trigger downstream signaling. NF-κB activation following IκBα Trim-Away was not indirectly due to TRIM21 activation (McEwan et al., 2013), because Trim-Away of a control endogenous protein, mTOR, triggered only minor NF-κB activity (Figures 6D and 6E). Electroporation of anti-IκBα antibody alone triggered a small induction of NF-κB, although to a much lesser extent than in cells expressing mCherry-TRIM21 (Figures 6D and 6E). It is possible that this small induction by anti-IκBα antibody alone was due to minor disruption of the NF-κB-IκBα interaction, because IκBα protein was not degraded in this condition (Figure 6E).

Collectively, these data establish that Trim-Away can be used to perform rapid protein depletion in bulk cell populations upon electroporation of specific antibodies. Furthermore, Trim-Away allows specific gain-of-function and loss-of-function phenotypes to be observed within just a few hours.

### Co-electroporation of TRIM21 Protein and Antibody Facilitate Rapid Protein Degradation in Unmodified Cell Lines

**Figure 7:**
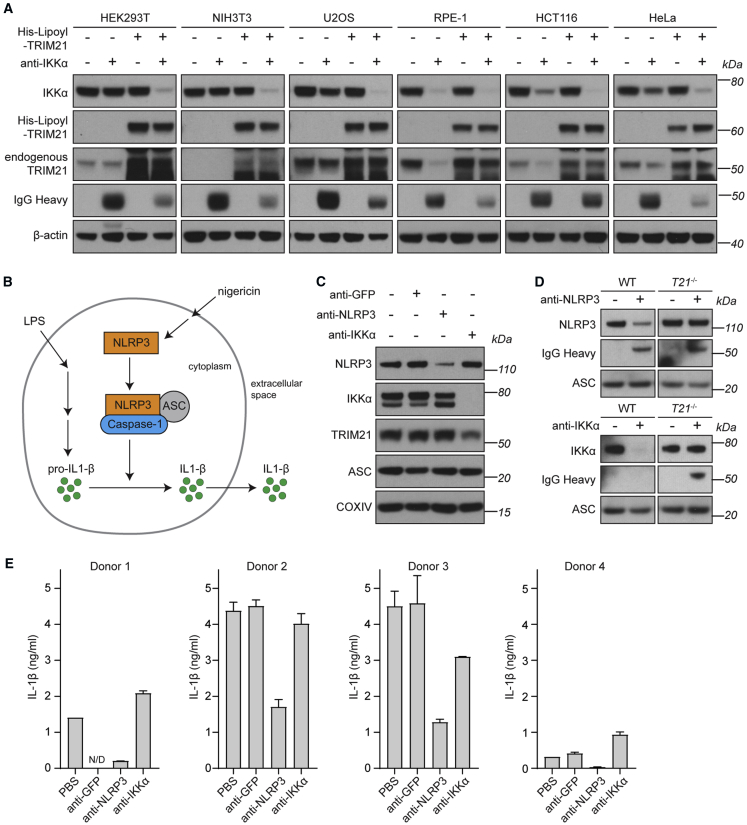
Trim-Away in Unmodified Cells and Primary Human Macrophages (A) The indicated cell lines were electroporated with PBS, anti-IKKα antibody, His-Lipoyl-TRIM21, or anti-IKKα + His-Lipoyl-TRIM21 and whole cell lysates harvested 3 hr later for immunoblotting. (B) Schematic of NLRP3 function. (C) HMDMs were electroporated with PBS, anti-GFP, ant-NLRP3, or anti-IKKα antibodies, then stimulated with 10 ng/mL LPS for 4 hr before collecting whole cell lysates for immunoblotting. (D) Bone marrow-derived macrophages (BMDMs) from wild-type (WT) or *Trim21* knockout (*T21*^−/−^) mice were electroporated with PBS, ant-NLRP3, or anti-IKKα antibodies, then stimulated with 10 ng/mL LPS for 4 hr before collecting whole cell lysates for immunoblotting. (E) HMDMs from 4 different blood donors were electroporated with PBS, anti-GFP, ant-NLRP3, or anti-IKKα antibodies and assayed for inflammasome activation (STAR Methods). Mean IL-1β values taken from 2 replicates for each condition are shown for each donor. Error bars show SD. See also Figures S4, S6, and S7.

**Figure S7:**
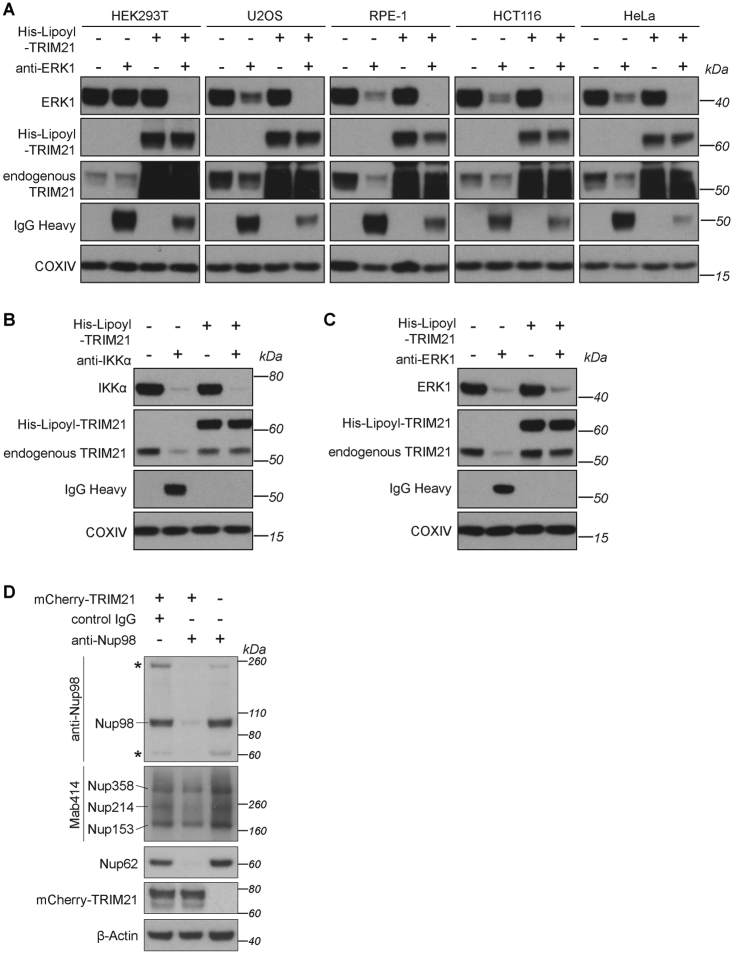
Requirements for Trim-Away Efficiency and Specificity, Related to Figure 1, Figure 2, Figure 3, Figure 4, Figure 5, Figure 6, Figure 7 (A) The indicated cell lines were electroporated with PBS, anti-ERK1 antibody, His-Lipoyl-TRIM21, or anti-ERK1 + His-Lipoyl-TRIM21 and whole cell lysates harvested 3 hours later for immunoblotting. (B and C) Normal human lung fibroblasts (NHLFs) were electroporated with the indicated proteins/antibodies and whole cell lysates harvested 3 hours later for immunoblotting. (D) HEK293T and HEK293T-mCherry-TRIM21 cell lines were electroporated with control IgG or an antibody directed against the Nucleoporin Nup98 (anti-Nup98), harvested 3 hours later and lysates immunoblotted for the indicated proteins. The Nup98 antibody was raised against the Nup98 N terminus, which is rich in phenylalanine-glycine (FG) repeats. The FG-repeats are shared by several other nucleoporins collectively known as the FG Nups (Brohawn et al., 2009). Asterisks show additional bands recognized by the anti-Nup98 antibody which are the same size as Nup214 and Nup62 recognized by the Mab414 and anti-Nup62 antibodies respectively. Consistent with non-specific binding, Trim-Away using the anti-Nup98 N-terminal antibody also triggered degradation of Nup62 and Nup214. Other nucleoporins Nup358 and Nup153 were not degraded, suggesting that co-depletion was not the result of degradation of the entire nuclear pore complex.

While Trim-Away performed efficiently when TRIM21 is ectopically overexpressed, this requires prior modification of a cell line. We therefore investigated whether TRIM21 could be supplied exogenously in protein form, eliminating the necessity for prior transfection or transduction. We envisaged that Trim-Away could be achieved in one step by co-electroporating antibody together with recombinant TRIM21 protein. To this end, we established a protocol to purify functional recombinant TRIM21 (STAR Methods). We tested this method by co-electroporating recombinant TRIM21 and antibodies against IKKα or ERK1 into six commonly used cell lines. Remarkably, in all cell lines, this led to the efficient degradation of IKKα or ERK1 (Figures 7A and S7A). Interestingly, in some cell lines, IKKα and ERK1 were at least partially degraded when antibody was electroporated without recombinant TRIM21 (Figures 7A and S7A). This degradation was linked to a marked decrease in the levels of endogenous TRIM21, suggesting that the endogenous TRIM21 was mediating protein degradation.

These data show that Trim-Away can be used to rapidly degrade endogenous proteins in unmodified cells by simply electroporating antibody and TRIM21 protein simultaneously. It also shows that some cell lines have sufficient endogenous active TRIM21 to mediate protein degradation, although this is likely to be dependent upon target expression levels.

### Trim-Away by Endogenous TRIM21 in Human Primary Cells

The possibility to directly degrade endogenous proteins by Trim-Away is potentially transformative for studies of primary human cells, in which gene editing and RNAi are challenging. We therefore tested if we can use Trim-Away to degrade proteins in normal human lung fibroblasts (NHLFs), primary cells used to study respiratory infection. Co-electroporation of IKKα or ERK1 antibodies together with recombinant TRIM21 led to efficient protein degradation in these cells (Figures S7B and S7C). Even endogenous levels of TRIM21 were sufficient to mediate protein degradation, as suggested by concomitant degradation of TRIM21 together with the target protein upon antibody electroporation alone (Figures S7B and S7C).

Studying protein function in primary human immune cells has long represented a difficult challenge (Chow et al., 2011). This is particularly true for primary macrophages, as their active nucleotide sensing machinery makes classical plasmid and siRNA transfection approaches unfeasible and likely to indirectly induce stimulation (Hornung and Latz, 2010). Because Trim-Away acts exclusively at the protein level, this method offers a unique opportunity to investigate protein function in primary human macrophages without perturbing them. NLRP3 is an intracellular signaling molecule expressed in immune cells that triggers the formation of a multiprotein complex called the inflammasome in response to diverse pro-inflammatory stimuli. The inflammasome directs interleukin-1β (IL-1β) maturation and secretion as part of a linked inflammatory and cell death response called pyroptosis (Schroder and Tschopp, 2010). The requirement for the NLRP3 protein in inflammasome activation has so far only been shown in mouse macrophages (Sutterwala et al., 2006) and a human leukemia-derived THP-1 cell line (Papin et al., 2007). We used Trim-Away to test whether NLRP3 is required for inflammasome activation in *ex vivo* human monocyte-derived macrophages (HMDMs). Electroporation of anti-NLRP3 antibody into HMDMs triggered rapid depletion of NLRP3 (Figure 7C). This was likely TRIM21-dependent, because NLRP3 was degraded upon anti-NLRP3 antibody electroporation into macrophages derived from wild-type mice, but not in macrophages from TRIM21 knockouts (Figure 7D). We did not observe co-degradation of TRIM21 and anti-NLRP3 antibody (Figures 7C and 7D), presumably because they were present in large excess to endogenous NLRP3. Strikingly, Trim-Away of NLRP3 caused a reduction in IL1-β secretion in response to stimulation with lipopolysaccharides (LPS) and the pore-forming toxin Nigericin in HMDMs from all four blood donors (Figure 7E). Neither electroporation of an anti-GFP antibody nor Trim-Away of IKKα had any effect on IL1-β secretion (Figure 7E). These results demonstrate for the first time that NLRP3 has a nonredundant and crucial role in inflammasome activation in primary human macrophages. Moreover, taken together the above data demonstrate that Trim-Away is suitable to degrade proteins in a wide range of cell types, including primary human cells that are intractable to other methods.

### Specificity of Trim-Away Assays

The successful application of Trim-Away to 9 different endogenous proteins in 10 different cell types demonstrates that the Trim-Away method is widely applicable and cell and substrate independent. Importantly, Trim-Away did not lead to the degradation of proteins in close spatial proximity of the target proteins: Trim-Away of IκBα did not cause degradation of NF-κB (Figures 6D and 6E), Trim-Away of individual nucleoporins did not lead to degradation of the entire nuclear pore complex (Figure S7D), Trim-Away of pericentrin did not cause degradation of other centrosomal proteins (Figures 5F and S5M), and Trim-Away of H2B-GFP did not cause degradation of H2A (Figures S3D–S3G). However, the fate of multiprotein complexes following Trim-Away of individual components may depend on the biology of the complex in question. For example, rapid degradation of CENP-A using the auxin-inducible degron system also causes degradation of the CENP-A-interacting protein HJURP (Hoffmann et al., 2016). Similarly, degradation of mTOR by Trim-Away also led to a reduction of Raptor and Rictor, which form complexes with mTOR (Figures 6A and 6B).

The Trim-Away mechanism itself is highly specific, as shown in rescue experiments (Figures 3A, 3E, and 3F) and by using multiple antibodies raised against different regions of the same protein (Figures 5D–5F and S5I–S5M). These approaches can be readily employed to confirm the specificity of Trim-Away phenotypes. Non-specific antibodies should be avoided because Trim-Away with these antibodies can lead to the simultaneous degradation of several proteins (Figure S7D). Thus, the specificity of the Trim-Away approach will be determined by the antibody used.

## Discussion

Gene knockouts and RNAi are widely used methods to study protein function. However, both methods act indirectly by blocking the expression of a protein and require that the target protein turns over. This can result in delayed protein disruption, an accumulation of non-specific defects and the activation of compensatory mechanisms. Current methods that act directly at the level of the protein either require that the endogenous protein is first replaced by a modified protein variant or are only applicable to a very small number of proteins.

Here, we have developed a widely applicable technique to acutely and rapidly degrade endogenous proteins in mammalian cells without the need for prior modification of the protein-coding gene or mRNA (Figure 1A). To our knowledge, this is the first posttranslational protein knockdown approach that can be readily applied to almost any intracellular protein.

We have shown that Trim-Away can degrade diverse protein substrates in both mammalian cell culture and primary mouse and human cells. Importantly, because the TRIM21-antibody interaction is highly conserved and maintained both within and between species (Keeble et al., 2008), the Trim-Away approach should be applicable to any mammalian cell using antibodies produced in any mammalian species. Trim-Away may also facilitate loss-of-function experiments in species that are not genetically tractable such as various marine species, or mammals that have long generation times.

Given that antibodies have been used over decades for various assays that require native protein recognition such as immunoprecipitation or immunofluorescence there is a comprehensive resource of antibodies with confirmed specificity readily available for use in Trim-Away assays. The growing pool of recombinant nanobodies (Chirichella et al., 2017, Helma et al., 2015, Pleiner et al., 2015) can also be utilized for Trim-Away simply by fusion to the Fc domain of conventional antibodies to allow TRIM21 binding (Figures 2I–2K).

By utilizing specific antibodies, Trim-Away is also suitable for selectively degrading posttranslationally modified proteins, splice or mutant protein variants while preserving the unmodified/wild-type protein; an approach that is not possible using current DNA- and RNA-targeting methods. Trim-Away could also be applied to study the function of proteins in selected cellular compartments. For instance, proteins that function both in the cytoplasm but also in other membrane-enclosed compartments or organelles can now be studied selectively in the cytoplasm without perturbing their function elsewhere, where they are shielded from antibodies. Vice versa, one could consider to target antibodies, nanobody-Fc fusions or TRIM21 to specific cellular sites for the local degradation of proteins.

Our observation that the endogenous levels of TRIM21 are sufficiently high for protein degradation in several cell types demonstrates that Trim-Away may be applicable without introducing excess TRIM21. This further simplifies the application of Trim-Away, especially in primary cells. If endogenous TRIM21 levels are insufficient for protein degradation, antibodies can easily be co-electroporated with recombinant TRIM21 protein to facilitate the complete degradation of target proteins.

In summary, this study provides unprecedented tools for studying protein function. First, it allows protein function to be studied in non-dividing primary cells where DNA- and RNA-targeting methods are not suitable. Second, it allows the functional analysis of long-lived proteins that are resistant to current knockdown methods that rely on protein turnover. Third, removal of essential endogenous proteins can now be achieved without the introduction of protein modifications such as degrons. Fourth, the remarkable speed of Trim-Away means that phenotypes can be observed immediately following degradation of the endogenous protein at any stage of a particular biological process. Finally, aberrant protein expression or activation is a hallmark of many human diseases such as neurodegeneration (Aguzzi and O’Connor, 2010) and cancer (Hanahan and Weinberg, 2011). It may become possible in the future to adapt the Trim-Away method to develop novel therapeutics that target disease-causing proteins for degradation.

## STAR★Methods

### Key Resources Table

**Table:** 

REAGENT or RESOURCE	SOURCE	IDENTIFIER
**Antibodies**		

Rabbit ant-GFP	Abcam	Cat#ab6556; RRID_AB305564
Mouse anti-GFP (9F9.F9)	Abcam	Cat#ab1218; RRID:AB_298911
Rabbit anti-Eg5	Sigma	Cat#HPA010568; RRID:AB_1848034
Rabbit anti-Rec8	Maureen Eijpe	https://doi.org/10.1083/jcb.200212080
Mouse anti-polyglutamine (3B5H10)	Sigma	Cat#P1874; RRID:AB_532270
Normal Rabbit IgG	Millipore	Cat#12-370; RRID:AB_145841
Normal Mouse IgG	Millipore	Cat#12-371; RRID:AB_145840
Rabbit anti-Pericentrin	Abcam	Cat#ab4448; RRID:AB_304461
Mouse anti-Pericentrin (clone 30)	BD Biosciences	Cat#611815; RRID:AB_399295
Rabbit anti-mTOR (7C10)	Cell Signaling Technology	Cat#2983; RRID:AB_2105622
Rabbit anti-IκBα (E130)	Abcam	Cat#ab215972; RRID:AB_733068
Rat anti-Nup98 (2H10)	Abcam	Cat#ab50610; RRID:AB_881769
Mouse anti-Eg5 (10C7)	Novus Biologicals	Cat#NB100-78467; RRID:AB_1084423
Rabbit anti-mCherry	Abcam	Cat#ab167453; RRID:AB_2571870
Rabbit anti-Cdk5rap2	Millipore	Cat#ABE236; RRID:AB_10918303
Mouse anti-γ-tubulin (GTU-88)	Sigma	Cat#T6557; RRID:AB_477584
Rabbit anti-Pcm1	Sigma	Cat#HPA023374; RRID:AB_1855073
Rabbit anti-Raptor	Millipore	Cat#09-217; RRID:AB_612103
Rabbit anti-S6 ribosomal protein (5G10)	Cell Signaling Technology	Cat#2217; RRID:AB_331355
Rabbit anti-phospho-S6 ribosomal protein ser235/236 (D57.2.2E)	Cell Signaling Technology	Cat#4858; RRID:AB_916156
Rabbit anti-Rictor (53A2)	Cell Signaling Technology	Cat#2114; RRID:AB_2179963
Rabbit anti-Akt (C67E7)	Cell Signaling Technology	Cat#4691; RRID:AB_915783
Rabbit anti-phospho-Akt ser473 (D9E)	Cell Signaling Technology	Cat#4060; RRID:AB_2315049
Mouse anti-IκBα (L35A5)	Cell Signaling Technology	Cat#4814; RRID:AB_390781
Rabbit anti-NFκB p65 (D14E12)	Cell Signaling Technology	Cat#8242; RRID:AB_10859369
Rabbit anti-ERK1 (Y72)	Abcam	Cat#ab214168; RRID:AB_732202
Rabbit anti-IKKα (Y463)	Abcam	Cat#ab59415; RRID:AB_733070
Mouse anti-NLRP3 (Cryo-2)	Adipogen	Cat#AG-20B-0014; RRID:AB_2490202
Rabbit anti-ASC (AL177)	Adipogen	Cat#AG-25B-0006; RRID:AB_2490440
Rabbit anti-IRF-3	Millipore	Cat#07-2193; RRID:AB_10807579
Mouse anti-Nuclear Pore Complex Proteins (Mab414)	Abcam	Cat#ab24609; RRID:AB_448181
Rabbit anti-Nup62	Abcam	Cat#ab96134; RRID:AB_10687293
Mouse anti-β-actin HRP-conjugated	Santa Cruz	Cat#sc-47778 HRP; RRID:AB_626632
Rat anti-tyrosinated-α-tubulin (YOL1/34)	AbD Serotec	Cat#MCA78G; RRID:AB_325005
Rabbit anti-COXIV	LI-COR	Cat#926-42214
Rabbit anti-Histone H2A	Abcam	Cat#ab18255; RRID:AB_470265
Mouse IgG1, kappa monoclonal (MG1-45)	Abcam	Cat#ab18447
Donkey anti-Goat IgG (H+L) Cross-Adsorbed Secondary Antibody, Alexa Fluor 488	ThermoFisher	Cat#A11055; RRID:AB_2534102
Goat anti-Rabbit IgG (H+L) Highly Cross-Adsorbed Secondary Antibody, Alexa Fluor 488	ThermoFisher	Cat#A11034; RRID:AB_2576217
Goat anti-Rabbit IgG (H+L) Highly Cross-Adsorbed Secondary Antibody, Alexa Fluor 647	ThermoFisher	Cat#A21245; RRID:AB_2535813
Goat anti-Mouse IgG (H+L) Highly Cross-Adsorbed Secondary Antibody, Alexa Fluor 647	ThermoFisher	Cat#A21236; RRID:AB_2535805
Rabbit anti-Mouse IgG HRP-conjugated	Dako	Cat#P0260; RRID:AB_2636929
Goat anti-Rabbit IgG (H+L) Cross-Adsorbed Secondary Antibody, HRP	ThermoFisher	Cat#31462; RRID:AB_228338
Mouse anti-Rabbit light chain specific,HRP cojugated	Millipore	Cat#MAB201P; RRID:AB_827270
Goat anti-Mouse light chain specific, HRP conjugated	Millipore	Cat#AP200P; RRID:AB_805324

**Biological Samples**		

Human whole blood samples (ethical approval REC08/H0308/176)	National Blood Service	Cat#NC07 (Buffy Coats 50ml)

**Chemicals, Peptides, and Recombinant Proteins**		

Hoechst 33342	ThermoFisher	Cat#H3570
MG132 (Z-Leu-Leu-Leu-al)	Sigma	Cat#C2211
Rapamycin	Cell Signaling Technology	Cat##9904
dbcAMP (N6,2′-O-Dibutyryladenosine 3′,5′-cyclic monophosphate sodium salt)	Sigma	Cat#D0627
Monastrol	Sigma	Cat#M8515
His-Lipoyl-hTRIM21	This paper	N/A
Recombinant Human M-CSF	R&D Systems	Cat#216-MC
Lipopolysaccharides (LPS)	Sigma	Cat#L4391
Nigericin	Enzo Life Sciences	BML-CA421-0005

**Critical Commercial Assays**		

Neon transfection system	ThermoFisher	Cat#MPK5000S
Steadylite plus Reporter Gene Assay System	Perkin Elmer	Cat#6066756
mMESSAGE mMACHINE T7 Transcription Kit	ThermoFisher	Cat#AM1334
Human IL-1β/IL-1F2 DuoSet ELISA	R&D Systems	Cat#DY201

**Deposited Data**		

RNA sequencing data	This paper	GEO: GSE105471

**Experimental Models: Cell Lines**		

Mouse: NIH 3T3	ATCC	Cat#CRL-1658; RRID:CVCL_0594
Mouse: NIH 3T3-mCherry-mTRIM21	This paper	N/A
Human: HEK293T	ATCC	Cat#CRL-1573; RRID:CVCL_0045
Human: HEK293T-mCherry-hTRIM21	This paper	N/A
Human: U2OS	ATCC	Cat#HTB-96; RRID:CVCL_0042
Human: RPE-1	ATCC	Cat#CRL-4000,: RRID:CVCL_4388
Human: HCT116	ATCC	Cat#CCL-247; RRID:CVCL_0291
Human: HeLa	ATCC	Cat#CCL-2; RRID:CVCL_0030
Human: NHLF	Lonza	Cat#CC-2512

**Experimental Models: Organisms/Strains**		

Mouse: FVBn	Charles River	RRID:IMSR_CRL:207
Mouse: C57BL/6J	The Jackson Laboratory	Cat#000664; RRID:IMSR_JAX:000664
Mouse: C57BL/6-*Trim21*^*tm1Hm*^/J	The Jackson Laboratory	Cat#010724; RRID:IMSR_JAX:010724

**Recombinant DNA**		

pmCherry-C1	Clontech	Cat#632524
pmCherry-C1-*mTrim21*	This paper	N/A
pmCherry-C1-*mTrim21ΔRING-Box*	This paper	N/A
pmCherry-C1-*mTrim21ΔPRYSPRY*	This paper	N/A
pmEGFP-C3	Clontech	Cat#632482
pGEMHE	Liman et al., 1992	PMID:1419000
pGEMHE-mEGFP	This paper	N/A
pGEMHE-mCherry	This paper	N/A
pGEMHE-mEGFP-*mTrim21*	This paper	N/A
pGEMHE-mEGFP-*mTrim21ΔRING-Box*	This paper	N/A
pGEMHE-mEGFP-*mTrim21ΔPRYSPRY*	This paper	N/A
pGEMHE-mCherry-*mTrim21*	This paper	N/A
pGEMHE-mCherry-*mTrim21ΔRING-Box*	This paper	N/A
pGEMHE-mCherry-*mTrim21ΔPRYSPRY*	This paper	N/A
pSMPP	This paper	N/A
pSMPP-mCherry-*mTrim21*	This paper	N/A
pSMPP-mCherry-*hTRIM21*	This paper	N/A
HLTV	Mark Allen	N/A
HLTV-*hTRIM21*	David Jacques	N/A
pGEMHE-membrane-mEGFP	This paper	N/A
pGEMHE-NLS-mEGFP	This paper	N/A
pGEMHE-*H2B*-mEGFP	This paper	N/A
pGEMHE-*H2B*-mCherry	This paper	N/A
pGEMHE-vhhGFP4-hIgG1-Fc	This paper	N/A
pGEMHE-mEGFP-*EG5*	Clift and Schuh., 2015	https://doi.org/10.1038/ncomms8217
pGEMHE-mCherry-*MAP4*	Clift and Schuh., 2015	https://doi.org/10.1038/ncomms8217
pGEMHE-mEGFP-*Map4*	This paper	N/A
pEGFP-Q23	Narain et al., 1999	Addgene Plasmid #40261
pEGFP-Q74	Narain et al., 1999	Addgene Plasmid #40262
pcDNA3_NSlmb-vhhGFP4	Caussinus et al., 2011	Addgene Plasmid #35579
pCRV-Gag-Pol	Stuart Neil	N/A
pMD2.G	Didier Trono	Addgene Plasmid #12259
pGL4.32[*luc2P*/NF-κB-RE/Hygro]	Promega	Cat#E8491

**Software and Algorithms**		

Prism	GraphPad Software	N/A
FlowJo	FLOWJO, LLC	N/A
ImageJ	NIH	N/A
Imaris	BITPLANE	N/A

**Other**		

Zeiss LSM710 microscope	Zeiss	N/A
Zeiss LSM780 microscope	Zeiss	N/A
Zeiss LSM800 microscope	Zeiss	N/A
Zeiss LSM880 microscope	Zeiss	N/A
AKTA Pure chromatography system	GE Healthcare	N/A
Neon transfection system	ThermoFisher	Cat#MPK5000S

### Contact for Reagent and Resource Sharing

Further information and requests for resources and reagents should be directed to and will be fulfilled by the lead contact, Melina Schuh (melina.schuh@mpibpc.mpg.de).

### Experimental Model and Subject Details

#### Mouse Strains

Female FVBn mice (8 weeks old) were obtained from a breeding colony that was generated using mice purchased from Charles River. C57BL/6J wild-type (WT) C57BL/6-*Trim21*^*tm1Hm*^/J (*T21*^*−/−*^) mice were obtained from The Jackson Laboratories. All mice were maintained in a specific pathogen-free environment according to UK Home Office regulations. The experiments involving mice have been approved by the MRC Cambridge Ethical Review Committee and the UK Home Office under project license numbers PPL 70/8087 and PPL PCF3F9520.

#### Cell lines

NIH 3T3, HEK293T, HCT116 and HeLa cells (ATCC) were cultured in DMEM medium (GIBCO; 31966021) supplemented with 10% Calf Serum and penicillin-streptomycin. U2OS cells (ATCC) were cultured in McCoy’s 5A (modified) medium (GIBCO; 36600021) supplemented with 10% Calf Serum and penicillin-streptomycin. RPE-1 cells (ATCC) were cultured in DMEM/F-12 medium (GIBCO; 10565018) supplemented with 10% Calf Serum and penicillin-streptomycin. Primary normal human lung fibroblast (NHLF) cells (Lonza; CC-2512) were cultured in FBM medium (Lonza; CC-3131) with supplements (Lonza; CC4126) according to the manufacturer’s instructions. All cells were grown at 37°C in a 5% CO_2_ humidified atmosphere and regularly checked to be mycoplasma-free. The sex of NIH 3T3 and HCT116 cell lines is male. The sex of HEK293T, U2OS, RPE-1 and HeLa cells is female. The sex of NHLF cells was not disclosed (Lonza; CC-2512). For live imaging, NIH 3T3 cells were plated on 35 mm glass-bottom dishes (MatTek) and culture medium was replaced with CO_2_-independent medium (GIBCO; 18045054) supplemented with 10% Calf Serum, GlutaMAX (GIBCO; 35050061) and penicillin-streptomycin. Following electroporation, cells were grown in medium supplemented with 10% Calf Serum without antibiotics. For the proteasome inhibition experiment (Figures 1D and 1E), 25 μM MG132 (Sigma; C2211) or a corresponding amount of DMSO was added to the medium 90 minutes prior to the experiment. For the mTORC1 inhibition experiments (Figure 6B), cells were treated with 10 nM Rapamycin (CST-9904) or a corresponding amount of DMSO immediately following electroporation.

#### Primary mouse oocytes

Oocytes were isolated from ovaries of 8-week-old FVBn female mice and cultured in M2 medium covered by mineral oil at 37°C. Isolated oocytes were maintained in prophase arrest by addition of 250 μM dbcAMP (Sigma; D0627). To induce resumption of meiosis, oocytes were released into dbcAMP-free medium. For the Eg5 inhibition experiments (Figure 3) oocytes were treated with 100 μM monastrol (Sigma; M8515) or a corresponding amount of DMSO.

#### Primary human monocyte derived macrophages

Human whole blood samples (ethical approval REC 16/LO/0997) were obtained from the National Health Service Blood and Transplant, Cat no: NC07 (Buffy Coats 50ml). Information about sex, gender, and age of respective donors were blinded. Human Peripheral Blood Mononuclear cells (PBMCs) were purified from buffy coats by centrifugation over a Ficoll-Paque density gradient (GE Healthcare). CD14+ monocytes were further purified by positive selection using CD14 microbeads (Miltenyi Cat no. 130-050-201). CD14+ monocytes were plated in 6-well dishes with 2 × 10^6^ cells per well and differentiated for 7 days into macrophages in RPMI medium (GIBCO; 11876093) supplemented with 10% Calf Serum, penicillin-streptomycin and 40 ng/ml recombinant human MCSF (R&D Systems, 216-MC-025). Macrophages were harvested by incubating cells in ice cold PBS with 2% calf serum and 5 mM EDTA for 10 minutes at 4°C and scraping.

#### Primary mouse bone marrow derived macrophages

Primary bone marrow derived macrophages (BMDMs) were obtained by culturing bone marrow flushed from the femurs and tibias of 6-8 week old male C57BL/6J wild-type (WT) or C57BL/6-*Trim21*^*tm1Hm*^/J (*T21*^*−/−*^) mice in DMEM media supplemented with 10% Calf Serum, penicillin-streptomycin and 40ng/ml recombinant human MCSF (R&D Systems, 216-MC-025). After 6 days of differentiation, macrophages were harvested by incubating cells in ice cold PBS with 2% calf serum and 5 mM EDTA for 10 minutes at 4°C and scraping.

### Method Details

#### Protein expression constructs

The mouse *Trim21* coding sequence was cloned from cDNA isolated from C57BL/6 mouse embryonic fibroblasts. The human *TRIM21* coding sequence was cloned from cDNA isolated from HeLa cells. Sequences encoding amino acids 124-462 of mouse TRIM21 lacking the RING and B Box domains (*Trim21ΔRING-Box*) and amino acids 1-281 of mouse TRIM21 lacking the PRYSPRY domain (*Trim21ΔPRYSPRY*) were generated by PCR on the full length *Trim21* coding sequence. For transient expression in NIH 3T3 cells, the *Trim21*, *Trim21ΔRING-Box* and *Trim21ΔPRYSPRY* coding sequences were inserted into pmEGFP-C1 and pmCherry-C1 vectors (Clontech). For *in vitro* mRNA transcription and expression in oocytes, the *Trim21*, *Trim21ΔRING-Box* and *Trim21ΔPRYSPRY* sequences were inserted with or without N-terminal mEGFP or mCherry (Clontech) into the pGEMHE (Liman et al., 1992) plasmid. For lentivirus production the mouse *Trim21* and human *TRIM21* coding sequences were cloned with N-terminal mCherry (clonetech) into lentiviral vector pSMPP to generate pSMPP-mCherry-*Trim21* and pSMPP-mCherry-*TRIM21*. For recombinant protein production the human *TRIM21* coding sequence was cloned in frame with the His-Lipoyl tag in HLTV vector (Mark Allen) to generate HLTV-*hTRIM21*. To generate pGEMHE-mEGFP and pGEHME-mCherry, sequences encoding mEGFP and mCherry were inserted into pGEMHE. To generate membrane-anchored GFP (pGEMHE-membrane-mEGFP) a sequence encoding the first 15 amino acids of Gnai2 (MGCTVSAEDKAAAER) containing N-myristol and S-palmitoyl motifs was inserted N-terminal to mEGFP in pGEMHE-mEGFP. To generate nuclear-localized GFP (pGEMHE-NLS-mEGFP) a sequence encoding the nuclear-localization signal (NLS) from Nucleoplasmin (KRPAATKKAGQAKKKK) was inserted N-terminal to mEGFP in pGEMHE-mEGFP. To generate GFP nanobody fused to Fc (pGEMHE-vhhGFP4-hIgG1-Fc) the vhhGFP4 nanobody coding sequence (Addgene plasmid #35579) (Caussinus et al., 2011) was inserted N-terminal to the hIgG1-Fc coding sequence (pFuse-hIgG1-Fc1; Invivogen) into pGEMHE. The previously published coding sequences were inserted into pGEMHE together with mEGFP or mCherry to generate pGEMHE-*H2B*-mEGFP (Kanda et al., 1998), pGEMHE-*H2B*-mCherry (Kanda et al., 1998), pGEMHE-mCherry-*MAP4* (Olson and Olmsted, 1999) and pGEMHE-mEGFP-*EG5* (Kaseda et al., 2012). To generate pGEMHE-mEGFP-*Map4*, the mouse *Map4* coding sequence was amplified from cDNA isolated from mouse C2C12 cells and inserted with N-terminal mEGFP into pGEMHE. The EGFP-Htt74Q (mutant huntingtin) and EGFP-Htt23Q (normal huntingtin) constructs were a gift from David Rubinsztein (Addgene plasmids #40261 and #40262) and described previously (Narain et al., 1999). For *in vitro* mRNA transcription, pGEMHE plasmids were linearized and capped mRNA was synthesized with T7 polymerase (Ambion mMessage mMachine T7 kit) according to manufacturer’s instructions.

#### Transient protein expression

For expression in NIH 3T3 cells, plasmids were transfected using Fugene 6 (Promega) according to manufacturer’s instructions 18-24 hours prior to live imaging. For expression in oocytes, *in vitro* transcribed mRNA was microinjected into prophase-arrested oocytes as described previously (Schuh and Ellenberg, 2007). For TRIM21 overexpression in oocytes, ∼6-8 picolitres of *Trim21* mRNA was microinjected at a needle concentration of ∼0.2 μg/μl. Following mRNA microinjection, oocytes were incubated at 37°C for at least 3 hours to allow for protein expression before release from dbcAMP or antibody microinjection. For the Eg5 rescue experiment (Figure 3), mRNA or a corresponding amount of water was microinjected into oocytes arrested in meiosis I with a monopolar spindle.

#### Stable cell lines

Lentivirus particles were collected from HEK293T supernatant 3 days after co-transfection of pSMPP-mCherry-*Trim21* or pSMPP-mCherry-*TRIM21* constructs with HIV-1 GagPol expressor pcRV1 (a gift from Dr. Stuart Neil) and pMD2G, a gift from Didier Trono (Addgene plasmid #12259). Transfection was performed with Fugene 6 (Promega). Supernatant was filtered at 0.45 μm before storage at −80°C. HEK293T and NIH 3T3 cells were transduced with lentivirus particles at multiplicity ∼0.1 transducing units per cell and selected using puromycin at 2.5 μg/ml from 48 hours post-transduction. Transduced cells expressing high levels of mCherry-TRIM21 exhibited aggregated protein that was non-functional. For NIH 3T3 cells, low expressing cells were therefore selected by flow cytometry (i-Cyt Synergy cell sorter) and a HEK293T single cell clone bearing disperse mCherry-TRIM21 was selected following limiting dilution. Expression of mCherry-TRIM21 in both cell lines was verified by flow cytometry using cells fixed in 4% formadehyde on a BD LSR II Flow Cytometer (Beckman Coulter). Flow cytometry data were analyzed using FlowJo software.

#### Protein Purification

*E. coli* C41 cells were transformed with HLTV-*hTRIM21* plasmid and His-Lipoyl-TRIM21 protein expression induced with 1 mM IPTG overnight at 18°C. Cleared cell lysates were prepared by sonication of cell pellets in 50 mM Tris pH 8, 150 mM NaCl, 1 mM DTT, 5 mM Imidazole pH 8, 20% (vol/vol) BugBuster (Novagen) and cOmplete protease inhibitors (Roche), followed by centrifugation 16,000 × g for 30 min. Lysates were loaded onto Ni-NTA agarose (QIAGEN), and His-Lipoyl-TRIM21 protein eluted with 300 mM Imidazole. Eluted protein was passed through a HiLoad 26/600 Superdex 200 PG size exclusion column using an ÄKTA pure purification system (GE Healthcare). Peak fractions were pooled and concentrated using Amicon Ultra-4 centrifugal filter units (Millipore) to a concentration of 6 mg/ml and dialysed in PBS for 2 × 2 hours using Slide-A-Lyzer Dialysis Cassettes (Thermo Fisher). His-Lipoyl-TRIM21 protein aliquots were frozen and stored at −80°C.

#### Antibody microinjection

The anti-GFP antibody used was rabbit anti-GFP (Abcam ab6556). The anti-Eg5 antibody used was rabbit anti-Eg5 (Sigma HPA010568). The anti-Rec8 antibody used was a gift from Maureen Eijpe (Eijpe et al., 2003). The mouse anti-polyglutamine 3B5H10 antibody was from Sigma (P1874). The control IgG used was normal rabbit IgG (Millipore 12-370). With the exception of anti-Rec8, all antibodies were concentrated using Amicon Ultra-0.5 100 KDa centrifugal filter devices (Millipore) to remove traces of azide and replace the buffer with PBS. Prior to microinjection into mouse oocytes, antibodies were diluted in PBS containing 0.05% NP40 to the following concentrations: anti-GFP (0.73 mg/ml), anti-Eg5 (0.08 mg/ml), control IgG (0.73mg/ml and 0.08 mg/ml), anti-Rec8 (concentration unknown), anti-polyglutamine 3B5H10 (5 mg/ml). Antibody microinjection into mouse oocytes was performed as described for mRNA microinjection (Schuh and Ellenberg, 2007). Prior to microinjection into NIH 3T3 cells, antibodies were diluted in PBS to 0.5 mg/ml (anti-GFP) or 4.2 mg/ml (3B5H10) and spun at 14,000 rpm for 15 minutes at 4°C. Antibody microinjection into NIH 3T3 cells was performed using a Narishige micromanipulator and Eppendorf Femtojet system mounted on a Zeiss LSM710 confocal microscope. In brief, pressure was applied so that there was a constant flow of antibody solution out of the needle, which was then transiently inserted into the cytoplasm until a slight swelling was observed indicating successful microinjection.

#### Antibody electroporation

All antibodies used for electroporation were either purchased in azide-free formats or passed through Amicon Ultra-0.5 100 KDa centrifugal filter devices (Millipore) to remove traces of azide and replace buffer with PBS. All antibodies were diluted to 0.5-1 mg/ml in PBS prior to electroporation. For some experiments, antibodies were mixed equal volumes with His-Lipoyl-TRIM21 protein (6 mg/ml) or corresponding amounts of PBS and incubated on ice for 30 minutes prior to electroporation. Antibodies used for electroporation were Alexa Fluor 488-labeled anti-goat (Molecular Probes A11055), normal rabbit IgG (Millipore 12-370), normal mouse IgG (Millipore 12-371), Mouse IgG1, kappa monoclonal (MG1-45, abcam, ab18447), rabbit anti-Pericentrin (abcam ab4448), mouse anti-Pericentrin (30, BD Biosciences 611815), rabbit anti-mTOR (7C10, carrier-free CST-2983BF), rabbit anti-IκBα (E130, abcam ab215972). rat anti-Nup98 (2H10, abcam ab50610), rabbit anti-ERK1 (Y72, abcam ab214168), rabbit anti-IKKα (Y463, abcam, ab169743), mouse anti-GFP (9F9.F9, abcam, ab1218) and mouse anti-NLRP3 (Cryo-2, Adipogen, AG-20B-0014). Antibody electroporation was performed using the Neon Transfection System (Thermo Fisher). Cells were washed with PBS and resuspended in Buffer R (Thermo Fisher) at a concentration of 8 × 10^7^ cells ml^-1^. For each electroporation reaction 8 × 10^5^ cells (10 μl) were mixed with 2 μl of antibody, antibody/TRIM21 or PBS. The mixture was taken up into a 10 μl Neon Pipette Tip (Thermo Fisher) and electroporated using the following settings: 1400V, 20 ms, 2 pulses. Electroporated cells were transferred to medium supplemented with 10% Calf Serum without antibiotics. To determine electroporation efficiency, cells were electroporated with Alexa Fluor-488-labeled IgG (Molecular Probes A11055) and fixed 3 hours after electroporation in 4% formaldehyde and levels of cell-associated Alexa Fluor 488-labeled IgG were monitored using a BD FACSCalibur Flow Cytometer (Beckman Coulter). Flow cytometry data were analyzed using FlowJo software.

#### RNA sequencing

NIH 3T3 and NIH 3T3-mCherry-mTRIM21 cells were resuspended in Buffer R (ThermoFisher) at a concentration of 3 × 10^7^ cells ml^-1^. For each electroporation reaction 3 × 10^6^ cells (100 μl) were mixed with 7.5 μg of antibody (ab18447), BSA or PBS. The mixture was taken up into a 100 μl Neon Pipette Tip (Thermo Fisher) and electroporated using the following settings: 1400V, 20 ms, 2 pulses. Electroporated cells were transferred to medium supplemented with 10% Calf Serum without antibiotics. 16 h later, cells were washed with PBS and lysed with Trizol (TRizol reagent, Thermo Fisher, 15596026). For phase separation, chloroform was added. Total RNA in the upper layer was precipitated with isopropanol and washed with 70% ethanol. The sample was DNase (Turbo DNase, Thermo Fisher, 40226) treated, and purified total RNA was phenol/chloroform extracted. The strand specific Illumina TruSeq RNA library preparation kit v2 with polyA enrichment was used to construct libraries from total RNA. Subsequently the Illumina NextSeq 500 platform and a 75 cycles high output v2 kit were used to sequence the libraries. The produced single-end reads which passed Illumina’s chastity filter were subject to de-multiplexing and trimming of Illumina adaptor residuals using Illumina’s real time analysis software (no further refinement or selection). Quality of the reads in fastq format was checked with the software FastQC (version 0.11.5). The splice aware RNA mapping software STAR (version 2.5.1) was used to map the reads to the reference genome. To count the uniquely mapped reads to annotated genes, the software htseq-count (HTSeq version 0.6.0) was used. Normalization of the raw counts and differential gene expression analysis was carried out with help of the software package DESeq2 (version 1.6.3). Libraries, sequencing and data analysis described in this section were performed by Microsynth AG (Balgach, Switzerland).

#### NFκB assay

HEK293T and HEK293T-mCherry-TRIM21 cells were transfected with pGL4.32[*luc2P*/NF-κB-RE/Hygro] NFκB luciferase reporter plasmid (Promega E8491) using Fugene 6 (Promega) according to manufacturer’s instructions. After 16-24 hours, cells were electroporated with antibodies or PBS and plated in a 96-well plate at a density of 2 × 10^4^ cells per well. Cells were incubated at 37°C for 5 hours before addition of Steadylite Plus luciferase reagent (Perkin Elmer) and analysis on a BMG Pherastar FS plate reader. Luciferase readout values of 4 replicates for each condition were normalized to the mean value for HEK293T cells electroporated with PBS to give fold NFκB induction.

#### Inflammasome assay

Primary human monocyte derived macrophages (HMDMs) were electroporated with antibodies or PBS and plated in a 96-well plate at a density of 7.5 × 10^4^ cells/well. After 1 hour, cells were stimulated with 10 ng/ml Lipopolysaccharides (LPS; Sigma L4391) for 2 hours. Cells were then transferred to serum-free medium and stimulated with 10 μM Nigericin (Enzo Life Sciences, BML-CA421-0005) for a further 2 hours. Cell-free supernatants were harvested, and IL-1β assayed by ELISA (R&D Systems, DY201) using a BMG Pherastar FS plate reader. Mean IL-1β values were taken from 2 replicates for each condition.

#### Confocal microscopy

For live imaging, images were acquired with a Zeiss LSM710 microscope equipped with BiG (Binary GaASP) detectors or Zeiss LSM880 or Zeiss LSM800 microscopes using a 40x C-Apochromat 1.2 NA water-immersion objective and a Zeiss environmental incubator box, which maintained oocytes and NIH 3T3 cells at 37°C without CO_2_. For fixed cells, images were acquired with a Zeiss LSM710 microscope or Zeiss LSM780 microscope equipped with a 63x C-Apochromat 1.2 NA oil immersion objective. In some images, shot noise was decreased with a Gaussian filter.

#### Immunofluorescence

Cells/oocytes were fixed for 30-60 min at 37°C in 100 mM HEPES (pH 7; titrated with KOH), 50 mM EGTA (pH 7; titrated with KOH), 2% formaldehyde (methanol free) and 0.2% Triton X-100. Fixed cells/oocytes were incubated in PBS with 0.1% Triton X-100 overnight at 4°C. Antibody incubations were performed in PBS, 3% BSA and 0.1% Triton X-100. Primary antibodies used were rabbit anti-Pericentrin (abcam ab4448; 1:200), rabbit anti-Cdk5rap2 (Millipore ABE236; 1:200), mouse anti-γ-tubulin (GTU88, Sigma T6557; 1:1000) and rabbit anti-Histone H2A (abcam ab18255; 1:200). Secondary antibodies used were Alexa Fluor 488-labeled anti-rabbit (Molecular Probes A11034; 1:400), Alexa Fluor 647-labeled anti-rabbit (Molecular Probes A21245; 1:400) and Alexa Fluor 647-labeled anti-mouse (Molecular Probes A21236; 1:400). DNA was stained with 5 mg ml^-1^ Hoechst 33342 (Molecular Probes H3570).

#### Immunoblotting

Oocytes were washed in PBS, resuspended in NuPAGE LDS Sample Buffer (Thermo Fisher) and heated at 95°C for 5 mins. Cell lines and primary macrophages were washed in PBS, lysed in RIPA buffer (CST-9806) supplemented with a protease inhibitor cocktail (Roche), spun at 14000G for 10 min and cleared lysates mixed with NuPAGE LDS Sample Buffer and heated at 95°C for 5 mins. Samples were run on NuPAGE 4%–12% Bis-Tris gels (Thermo Fisher) and transferred onto nitrocellulose membrane. Antibody incubations were performed in PBS with 5% milk and 0.1% Tween-20. Primary antibodies used were mouse anti-Eg5 (10C7, Novus NB100-78467, 1:500), rabbit anti-Eg5 (HPA010568; 1:500), rabbit anti-mCherry (Abcam ab167453; 1:10000), mouse anti-Pericentrin (30, BD Biosciences 611815; 1:1000), rabbit anti-Pericentrin (abcam ab4448; 1:2000), rabbit anti-Cdk5rap2 (Millipore ABE236; 1:500), mouse anti-γ-tubulin (GTU88, Sigma T6557; 1:2000), rabbit anti-PCM1 (Sigma HPA023374; 1:500), rabbit anti-mTOR (7C10, CST-2983; 1:1000), rabbit anti-Raptor (Millipore 09-217; 1:1000), rabbit anti-S6 ribosomal protein (5G10, CST-2217; 1:000), rabbit anti-phospho-S6 ribosomal protein ser235/236 (D57.2.2E, CST4858; 1:2000), rabbit anti-Rictor (53A2, CST-2114, 1:1000), rabbit anti-Akt (C67E7, CST-4691, 1:1000), rabbit anti-phospho-Akt ser473 (D9E, CST-4060), mouse anti-IκBα (L35A5, CST-4814; 1:1000), rabbit anti-IκBα (E130, abcam ab215972; 1:5000), rabbit anti-NFκB p65 (D14E12, CST-8242, 1:1000), rabbit anti-ERK1 (Y72, abcam ab214168; 1:10000), rabbit anti-IKKα (Y463, abcam ab169743; 1:5000), mouse anti-NLRP3 (Cryo-2, Adipogen AG-20B-0014; 1:500), rabbit anti-ASC (AL177, Adipogen AG-25B-000; 1:500), mouse anti-TRIM21 (D-12, Santa Cruz Biotechnology sc-25351; 1:500), rabbit anti-IRF-3 (Millipore 07-2193; 1:1000), rat anti-Nup98 (2H10, abcam ab50610; 1:1000), mouse anti-Nuclear Pore Complex Proteins (Mab414; abcam ab24609; 1:1000); rabbit anti-Nup62 (abcam ab96134; 1:1000), mouse anti-β-actin HRP-conjugated (Santa Cruz sc-47778 HRP; 1:1000) rat anti-tyrosinated-α-tubulin (YOL1/34, AbD Serotec MCA78G; 1:5000) and rabbit anti-COXIV (LI-COR 926-42214; 1:5000). HRP-coupled secondary anti-mouse (Dako P0260), anti-rabbit light chain specific (Millipore AP200P), anti-rabbit (Fisher 31462), anti-rabbit light chain specific (Millipore MAB201P) and anti-rat (Santa Cruz sc-2032) were detected by enhanced chemiluminescence (Amersham, GE Healthcare) and X-ray films.

### Quantification and Statistical Analyses

#### Measurement of GFP fluorescence

Three-dimensional time-lapse images were acquired using Zeiss’s MultiTime Series macro. For NIH 3T3 cells, 7 confocal z sections every 1.0 μm covering the entire cell were captured just before antibody microinjection and in 5 minute intervals from microinjection onward. For oocytes, 3 confocal z sections every 1.3 μm surrounding either the center of the cell (for free GFP and membrane-anchored GFP) or the maximum fluorescence (for H2B-GFP and nuclear-localized GFP) were captured just before antibody microinjection and in 2 minute intervals from microinjection onward. Using ImageJ software, the integrated density (the product of the area and mean intensity) was measured for the entire cell/oocyte from a single z section at each time point. GFP fluorescence was calculated by subtracting the product of the mean intensity of the background (a region outside the cell/oocyte) and the area of the cell/oocyte from the integrated density. GFP fluorescence values were normalized to the time point prior to antibody microinjection. For half-life measurements, Prism (GraphPad) software was used to fit single phase decay curves to the mean values of GFP fluorescence starting after the initial lag phase.

#### Immunofluorescence quantification

To quantify centrosomal Cdk5rap2, cells stained for Cdk5rap2 and γ-Tubulin were imaged (19 z-confocal sections every 0.5 μm). Centrosomes were segmented by applying a threshold in the γ-Tubulin channel using the isosurface function of Imaris (Bitplane) to generate a 3.5 μm diameter 3D centrosome region. The mean intensity for Cdk5rap2 within the centrosome region was measured using Imaris (Bitplane) and exported into Microsoft Excel. To quantify chromosomal H2B-GFP and H2A, oocytes expressing H2B-GFP and stained for histone H2A and DNA were imaged (single confocal section through the center of the chromosomes). Chromosomes were segmented by applying a threshold in the DNA channel using the isosurface function of Imaris (Bitplane). The mean intensity for H2B-GFP and H2A within the chromosome region was measured using Imaris (Bitplane) and exported into Microsoft Excel.

#### Statistical Analysis

Average (mean), standard deviation (s.d.) and statistical significance based on Student’s t test (two-tailed) for absolute values or Fisher’s exact test (two-tailed) for category values were calculated in Microsoft Excel or Graphpad Prism. All boxplots show median (line), mean (small square), 1^st^, 99^th^ (crosses), 5^th^, 95^th^ (whiskers) and 25^th^ and 75^th^ percentiles (boxes).

### Data and Software Availability

The accession number for the RNA sequencing data reported in this paper is GEO: GSE105471.

## Author Contributions

D.C. and M.S. conceived and designed the study. D.C. carried out all experiments, with the following exceptions: W.A.M. and D.C. optimized antibody electroporation. W.A.M. generated and characterized stable cell lines. L.I.L. and D.C. performed experiments with primary human and mouse macrophages. L.I.L. performed inflammasome assays. D.C. and L.C.J. purified recombinant TRIM21 protein. D.C. and V.K. performed selective huntingtin depletion experiments. V.K. performed RNA-seq experiments and analyses and analyzed cell behavior upon electroporation; B.M. performed the nanobody-Fc fusion experiment. D.C. and M.S. wrote the manuscript. L.C.J. edited the manuscript. M.S. supervised the study.

## Supplemental Information

Table S1. RNA Sequencing mCherry-TRIM21 versus Wild-Type (PBS), Related to Figure S5K

Table S2. RNA Sequencing mCherry-TRIM21 versus Wild-Type (BSA), Related to Figure S5L

Table S3. RNA Sequencing mCherry-TRIM21 versus Wild-Type (IgG), Related to Figure S5M

Movie S1. Rapid Degradation of GFP by Trim-Away, Related to Figure 1 An NIH3T3 cell expressing free GFP (green) and mCherry-TRIM21 (magenta) and was microinjected with anti-GFP antibody. Time shows minutes (min) from antibody microinjection, 0 min is just before antibody microinjection.

Movie S2. Eg5 Trim-Away Rescue Experiment, Related to Figure 3 An oocyte overexpressing TRIM21 and microinjected with anti-Eg5 antibody was allowed to form a monopolar spindle and then microinjected with *Eg5-mEGFP* mRNA. Microtubules were labelled with mCherry-MAP4. Time shows hours:minutes.

Movie S3. Rec8 Trim-Away Triggers Rapid Sister Chromatid Separation, Related to Figure 4 Oocytes overexpressing mEGFP-TRIM21 were allowed to progress to metaphase II arrest and then microinjected with either control IgG (left) or anti-Rec8 antibody (right). Chromosomes were labelled with H2B-mCherry.

Movie S4. Live Imaging of NIH3T3 Cells following Electroporation, Related to Figure S5 NIH3T3-mCherry-TRIM21 cells were taken up into the Neon Pipette Tip, either electroporated or not (mock), and imaged every 15 min for 70 hours.
